# Appendiceal Adenocarcinoma Cytoreduction Outcomes and Perioperative Serum Tumor Marker Levels

**DOI:** 10.1001/jamanetworkopen.2026.10569

**Published:** 2026-05-04

**Authors:** Vinay K. Pattalachinti, Ashlee Seldomridge, Abdelrahman Yousef, Mahmoud Yousef, Eleanor A. Fallon, Jacquelyn McCullough, Betul Beyza Gunes, Yun Song, Christopher P. Scally, Paul Mansfield, Michael G. White, Keith F. Fournier, Beth A. Helmink, John Paul Shen

**Affiliations:** 1 Department of Gastrointestinal Medical Oncology, The University of Texas MD Anderson Cancer Center, Houston; 2 The Joe R. and Teresa Lozano Long School of Medicine, University of Texas Health San Antonio, San Antonio; 3 Department of Surgical Oncology, University of Texas MD Anderson Cancer Center, Houston; 4 Department of Medicine, University of San Diego, La Jolla, California; 5 Department of Colon and Rectal Surgery, University of Texas MD Anderson Cancer Center, Houston

## Abstract

**Background:**

Appendiceal adenocarcinoma (AA) is a rare gastrointestinal cancer that frequently presents with peritoneal metastases; the standard of care for resectable peritoneal metastases from AA limited to the peritoneum is cytoreductive surgery (CRS) followed by hyperthermic intraperitoneal chemotherapy (HIPEC). Three serum tumor markers (TMs)—carcinoembryonic antigen (CEA), carbohydrate antigen 19-9 (CA19-9), and cancer antigen 125 (CA125)—may play a role in preoperative and postoperative risk estimation and decision-making in patients undergoing CRS-HIPEC.

**Objective:**

To evaluate the association of preoperative and postoperative serum TM levels with outcomes in patients with AA or goblet cell adenocarcinoma (GCA) undergoing CRS-HIPEC.

**Design, Setting, and Participants:**

This retrospective cohort study was conducted in a single quaternary referral cancer center. Software was used to query the MD Anderson internal patient database of patients with AA or GCA to identify a cohort receiving CRS with or without HIPEC between March 2016 and August 2024, with median postoperative follow-up of 35.6 months (range, 1.4-100.5 months). Data were analyzed from June 2024 to March 2025.

**Exposure:**

Preoperative and postoperative levels of CEA, CA19-9, and CA125.

**Main Outcomes and Measures:**

Association of preoperative and postoperative serum TM levels with tumor burden and likelihood of complete CRS, disease-free survival (DFS), and overall survival (OS).

**Results:**

A total of 421 CRSs were included in the study, 376 (89.3%) of which were performed in patients with AA (229 [60.9%] in females; median age at surgery, 56 [IQR, 47-64] years; 290 [77.1%] were complete CRS). In AA, greater vs lesser preoperative tumor burden was associated with increased median serum TM levels (CEA: 9.5 [IQR, 3.7-42.3] ng/mL vs 3.0 [IQR, 2.1-5.9] ng/mL; CA19-9: 31.1 [IQR, 10.0-102.3] U/mL vs 15.0 [IQR, 6.1-25.5] U/mL; CA125: 28.7 [IQR, 13.9-58.5] U/mL vs 11.5 [IQR, 8.0-17.3] U/mL; all *P* < .001), and after CRS vs before, percentages of patients with TM elevation were significantly lower (CEA: 77 of 223 [34.5%] vs 222 of 340 [65.3%]; CA19-9: 45 of 194 [23.2%] vs 117 of 324 [36.1%]; CA125: 13 of 203 [6.4%] vs 98 of 325 [30.2%]; all *P* < .001). However, complete vs incomplete CRS was associated with normalized TM levels in a greater percentage of patients (55 of 90 [61.1%] vs 7 of 52 [13.5%]; *P* < .001). Elevated vs normal preoperative TM levels were associated with increased rate of incomplete CRS (30 of 193 [15.5%] vs 1 of 95 [1.1%]; *P* < .001) and shorter DFS (hazard ratio [HR], 2.30; 95% CI, 1.46-3.64; *P* < .001) but not shorter OS (HR, 1.36; 95% CI, 0.71-2.60; *P* = .36). Postoperative TM elevation was associated with shorter DFS (HR, 3.73; 95% CI, 2.33-5.95; *P* < .001) and OS (HR, 4.10; 95% CI, 2.02-8.31; *P* < .001) in univariate analyses and also in multivariate analyses (HR, 3.34; 95% CI, 1.44-7.75; *P* = .005), whereas postoperative normalization of all TMs was associated with improved DFS (HR, 3.54; 95% CI, 2.01-6.23; *P* < .001) and OS (HR, 6.00; 95% CI, 2.06-17.46; *P* = .001) in univariate analyses and also in multivariate analyses (HR, 4.21; 95% CI, 1.33-13.35; *P* = .02).

**Conclusions and Relevance:**

In this cohort study of CRS in patients with AA, elevated serum TM levels were associated with worse outcomes, suggesting that patients with postoperative TM elevation after complete CRS may represent a cohort with residual tumor that requires more cautious surveillance and that both preoperative and postoperative levels of CEA, CA19-9, and CA125 should be assessed in clinical practice.

## Key Points

**Question:**

What is the clinical utility of preoperative and postoperative serum tumor marker (TM) levels in patients with appendiceal adenocarcinoma (AA) undergoing cytoreductive surgery (CRS)?

**Findings:**

In this cohort study of 376 CRSs, TM levels were associated with tumor burden and were significantly decreased following CRS. Elevated preoperative TM levels were associated with worse disease-free survival and decreased likelihood of complete CRS, while elevated TM levels and failure to normalize TM levels following CRS were associated with worse disease-free and overall survival.

**Meaning:**

The findings suggest preoperative and postoperative serum TM levels in patients with AA undergoing CRS may provide risk information and inform clinical decision-making.

## Teaser

This cohort study assesses whether elevated preoperative and postoperative serum tumor marker levels and postoperative normalization of these levels are associated with survival outcomes after cytoreductive surgery for appendiceal adenocarcinoma.

## Introduction

Appendiceal adenocarcinoma (AA) is a rare gastrointestinal cancer with an age-adjusted incidence of 1.3 per 100 000 persons in the US.^1^ AA metastases are typically confined to the peritoneal cavity, causing peritoneal carcinomatosis.^2^ Cytoreductive surgery (CRS) with hyperthermic intraperitoneal chemotherapy (HIPEC) is the standard of care for AA peritoneal metastases.^3^ CRS-HIPEC involves surgically resecting visible peritoneal disease before intraoperatively delivering heated cytotoxic chemotherapy—usually mitomycin C or oxaliplatin—into the peritoneal cavity.^4,5,6^ In both retrospective and prospective studies, patients with AA peritoneal metastases benefited from CRS-HIPEC.^3,7,8,9^

Serum tumor markers (TMs) are used in various cancers for prognostication and quantifying treatment response.^10,11^ The role of serum TMs in patients with metastatic AA, however, remains unclear. Currently, 3 TMs are typically used to track gastrointestinal cancers: carcinoembryonic antigen (CEA),^12^ carbohydrate antigen 19-9 (CA19-9),^13^ and cancer antigen 125 (CA125).^14^ CEA is physiologically expressed by fetal tissue and is expressed at low levels by noncancerous gastrointestinal tissue. The mechanism through which serum CEA increases in patients with gastrointestinal cancers is unclear.^15^ CA19-9 is a glycosylation pattern of the Lewis-a antigen that is highly expressed in some patients with cancer, especially in those with pancreatic adenocarcinoma.^16^ CA125 is expressed by peritoneal, pleural, and pericardial tissue and is elevated by various physiologic and pathologic processes that disturb these coelomic tissues, including endometriosis and peritoneal metastases.^17^ Previous work by our group and others has quantified the association of preoperative and/or postoperative TM elevation in CRS outcomes.^18,19,20,21,22,23,24^ In both retrospective and prospective studies, various combinations of TM elevation were associated with decreased overall survival (OS) and disease-free survival (DFS). The results of these studies, however, have been inconsistent, potentially because the relative rarity of AA leads to underpowered studies.

In this study, we used a previously established automated system for clinical record extraction^25^ to identify and study CRS performed in a cohort of unique patients with AA or goblet cell adenocarcinoma (GCA), stratified based on elevation of any of the 3 TMs to better represent how CEA, CA19-9, and CA125 are assessed and interpreted clinically: not as each TM individually but as a set. Our goal was to assess whether preoperative and/or postoperative elevation and normalization of the serum TMs were associated with tumor burden, complete CRS, DFS, and OS in patients undergoing CRS.

## Methods

### Cohort Generation

In this cohort study, the MD Anderson Cancer Center (MDACC) adapted version of the Palantir-Foundry software system was used to query the MD Anderson Gastrointestinal Medical Oncology database to identify patients with AA treated between March 2016 and October 2024.^25^ Eligible patients had a pathologic diagnosis of AA, including mucinous, colonic, and signet ring subtypes. Patients with GCA were analyzed separately. Histologic classification and grade were collected from patients’ pathology records. Pathologic diagnosis was determined by a team of expert pathologists in gastrointestinal cancers.^26^ These patients were then subset to patients who received CRS at MDACC from March 2016 to August 2024. This was determined by querying all operative notes using the *Current Procedural Terminology (CPT)* codes related to CRS (96446, 96547, 96548, 77605, 58957, 49203, 49204, 49205, and 49255) and confirming the procedure was performed by 1 of 5 surgeons (included C.P.S., P.M., K.F.F., B.A.H.) specializing in peritoneal surface malignant tumors. CRS-HIPEC was performed as described previously.^27,28^ Patients were excluded if there was less than 1 month of follow-up or if they had a histologic diagnosis of only low-grade appendiceal mucinous neoplasm, high-grade appendiceal mucinous neoplasm, or neuroendocrine tumor without concurrent adenocarcinoma.^29^ Patients with AA were then manually verified to have received CRS to ensure accuracy. This study was approved by the University of Texas MD Anderson Cancer Center institutional review board. A waiver of informed consent was granted in accordance with the US Code of Federal Regulations (45 CFR §46.116 [Common Rule]), as the study was of minimal risk to patients. This study followed the Strengthening the Reporting of Observational Studies in Epidemiology (STROBE) reporting guideline for cohort studies.

### Data Collection

Demographic data such as patient sex, age, date of diagnosis, date of last follow-up, and date of death were collected in an automated fashion. Patients’ race and ethnicity were self-reported and were assessed to better understand if these factors were associated with survival or disease outcomes; categories were African American or Black; Asian; Hispanic or Latino; non-Hispanic White (hereafter, *White*); and other (included Alaska Native, American Indian, Native Hawaiian, or Other Pacific Islander), unknown, or declined to answer. TM measurements, performed at MDACC, and HIPEC agent were similarly collected. Completeness of cytoreduction (CCR) score, peritoneal cancer index (PCI) as a measure of tumor burden (range, 0-39, with higher values indicating increased peritoneal disease burden), and palliative intent were collected from operative notes in a semiautomated fashion using regular expression and were manually validated. Complete CRS was defined as a CCR score of 0 to 1 on a scale from 0 to 3 (with lower scores indicating a more complete cytoreduction); incomplete CRS was defined as a CCR score of 2 to 3. These definitions are based on historical practice and prognostic data.^30,31^ Palliative intent was defined as cases where surgeons had no preoperative expectation of complete CRS. Histologic subtypes and tumor grade were collected from pathologist notes in a similar semiautomated fashion. Cases performed on nongoblet AA tumors were separated from GCA. Unless otherwise stated, analyses were performed on the cohort with nongoblet AA without GCA. In cases where AA tumors were assigned to multiple histologic subtypes in different notes, patients were assigned a single subtype based on the following priority: first, signet ring; second, mucinous; and third, colonic.

Unless otherwise indicated, the unit of analysis was each unique CRS. The main explanatory variables were preoperative and postoperative elevation of any TM. Preoperative TM levels were defined as the measurement closest to the date of CRS within 100 days. Postoperative TM levels were defined as the lowest level within 180 days after CRS. TM levels were defined as elevated if they were above the laboratory upper limit of normal (CEA ≥3 ng/mL, CA19-9 ≥35 U/mL, and CA125 ≥35 U/mL from March 2016 to March 2018 and CEA >3.8 ng/mL, CA19-9 >35 U/mL, and CA125 >38 U/mL from April 2018 to August 2024, as the TM reference normal changed in 2018).

Our main response variables were DFS and OS. DFS was defined as the time from complete CRS to when relapse was documented in either radiology reports or clinical notes. OS was determined by calculating the difference between the date of first CRS and date of death. The unit of analysis for OS was each patient.

### Statistical Analysis

All statistical analyses were performed from June 2024 to March 2025 using R, version 4.5.2 (R Project for Statistical Computing), in RStudio, version 2026.1.0.392 (RStudio, PBC). Unless otherwise stated, analyses were conducted using both palliative- and curative-intent CRS. Baseline characteristics of the study population were summarized by frequency and percentage. Differences between groups were compared using the χ^2^ test for categorical variables and Student *t* test or Wilcoxon signed-rank test for continuous variables. Time-to-event end points were visualized using Kaplan-Meier curves generated using the survival package in R.^32^ Differences in DFS and OS were compared using the Cox proportional hazards regression model or the log-rank test where appropriate. Two-sided *P* < .05 was used for significance throughout.

## Results

### Cohort Characteristics

Using the semiautomated method, we identified 421 CRSs performed on 377 unique patients (eFigure 1 in Supplement 1). Most CRSs (376 [89.3%]) were performed in patients with AA (229 [60.9%] in females and 147 [39.1%] in males, with median patient age at surgery of 56 years [IQR, 47-64 years]); 26 CRSs (6.9%) were performed in African American or Black patients, 12 (3.2%) in Asian patients, 50 (13.3%) in Hispanic or Latino patients, 280 (74.5%) in White patients, and 8 (2.1%) in patients with other or unknown race and ethnicity or who declined to answer (Table). In most of the 376 CRSs performed in patients with AA, the patients received HIPEC along with CRS (312 [83.0%]), with the most common agent being mitomycin C (242 of 312 patients [77.6%]). Of the CRSs in patients with AA, 290 (77.1%) were complete and 86 (22.9%) were incomplete or palliative-intent CRS. Median PCI for the entire cohort was 20 (IQR, 11-29), with lower PCI in cases of complete (18 [IQR, 9-25]) than incomplete (32 [IQR, 26-37]) CRS. More CRSs were performed on low-grade (well or well to moderately differentiated) tumors (245 [65.2%]) compared with high-grade (moderately or poorly differentiated) tumors (131 [34.8%]) (Table). Of the 335 patients with AA, 59 (17.6%) died during the study period. Median follow-up was 35.6 months (range, 1.4-100.5 months). Of 273 complete CRSs performed with follow-up imaging (94.1% of the 290 complete CRSs), relapse occurred in 121 (44.3%) during the study period, with a median follow-up with imaging of 20.0 months (range, 3.5-97.2 months).

**Table.** Cohort Information

Characteristic	CRS procedures among patients with AA, No. (%)		
	Complete (n = 290 [77.1])	Incomplete (n = 86 [22.9])	Total (N = 376 [100])
Patients, No./total No. (%)	263/335 (78.5)	82/335 (24.5)	335/335 (100)
Age, median (IQR), y			
At diagnosis	55 (46-62)	54 (47-62)	55 (47-62)
At surgery	57 (47-65)	56 (49-63)	56 (47-64)
Time from diagnosis to surgery			
<6 mo	119 (41.0)	32 (37.2)	151 (40.2)
6 mo to 2 y	70 (24.1)	23 (26.7)	93 (24.7)
>2 y	101 (34.8)	312 (36.0)	132 (35.1)
Sex			
Female	187 (64.5)	42 (48.8)	229 (60.9)
Male	103 (35.5)	44 (51.2)	147 (39.1)
Histologic subtype			
Mucinous	216 (74.5)	71 (82.6)	287 (76.3)
Signet	9 (3.1)	1 (1.2)	10 (2.7)
Adenocarcinoma, not otherwise specified	65 (22.4)	14 (16.3)	79 (21.0)
Survival after CRS			
Overall			
Median (95% CI), mo	NR	53.4 (35.7-NR)	NR
5 y, % (95% CI)	86.5 (81.6-91.6)	45.9 (31.9-66.2)	79.2 (74.0-84.6)
Disease-free			
Median (95% CI), mo	48.7 (33.3-NR)	NA	NA
5 y, % (95% CI)	47.1 (40.6-54.9)	NA	NA
Race and ethnicity			
African American or Black	11 (3.8)	15 (17.4)	26 (6.9)
Asian	9 (3.1)	3 (3.5)	12 (3.2)
Hispanic or Latino	39 (13.4)	11 (12.8)	50 (13.3)
Non-Hispanic White	224 (77.2)	56 (65.1)	280 (74.5)
Other, unknown, or declined to answer^a^	7 (2.4)	1 (1.2)	8 (2.1)
PCI, median (IQR)^b^	18 (9-25)	32 (26-37)	20 (11-29)
Tumor grade			
Well or well to moderately differentiated	186 (64.1)	59 (68.6)	245 (65.2)
Moderately or poorly differentiated	104 (35.9)	27 (31.4)	131 (34.8)
Microsatellite stability			
MSS	128 (100)	93 (100)	35 (100)
MSI-H	0	0	0
Procedures with TMs collected			
Preoperative			
CEA	271 (93.4)	79 (91.9)	350 (93.1)
CA19-9	251 (86.7)	73 (84.9)	324 (86.1)
CA125	250 (86.2)	75 (87.2)	325 (86.4)
Postoperative			
CEA	162 (55.9)	61 (70.9)	223 (59.3)
CA19-9	144 (49.7)	50 (58.1)	194 (51.6)
CA125	150 (51.7)	53 (61.6)	203 (54.0)
Preoperative and postoperative			
CEA	151 (52.1)	56 (65.1)	207 (55.1)
CA19-9	125 (43.1)	43 (50.0)	168 (44.7)
CA125	126 (43.4)	45 (52.3)	171 (45.5)
Repeat CRS			
First	260 (89.7)	75 (87.2)	335 (89.1)
Second	30 (10.3)	10 (11.6)	40 (10.6)
Third	0	1 (1.2)	1 (0.3)
ECOG performance status^c^			
0	134 (69.4)	37 (53.6)	171 (65.3)
1	55 (28.5)	26 (37.7)	81 (30.9)
2	4 (2.1)	2 (2.9)	6 (2.3)
3	0	4 (5.8)	4 (1.5)
HIPEC			
None	32 (11.0)	32 (37.2)	64 (17.0)
Mitomycin C	201 (69.3)	41 (47.7)	242 (64.4)
Oxaliplatin	30 (10.3)	3 (3.5)	33 (8.8)
Cisplatin	27 (9.3)	10 (11.6)	37 (9.8)

Abbreviations: AA, appendiceal adenocarcinoma; CA125, cancer antigen 125; CA19-9, carbohydrate antigen 19-9; CEA, carcinoembryonic antigen; CRS, cytoreductive surgery; ECOG, Eastern Cooperative Oncology Group; HIPEC, hyperthermic intraperitoneal chemotherapy; MSS, microsatellite stable; MSI-H, microsatellite instability high; NA, not applicable; NR, not reported; PCI, peritoneal cancer index; TM, tumor marker.

^a^ Other race included Alaska Native, American Indian, Native Hawaiian, or Other Pacific Islander.

^b^ Index range, 0 to 39, with higher values indicating increased peritoneal disease burden.

^c^ Scale range, 0 to 5, with lower scores indicating better functioning.

Of the 376 CRSs, 350 (93.1%) had preoperative CEA measurements, 324 (86.2%) had preoperative CA19-9, and 325 (86.4%) had preoperative CA125. Fewer CRSs had postoperative TM measurements, with 223 (59.3%) having postoperative CEA, 194 (51.6%) having postoperative CA19-9, and 203 (54.0%) having postoperative CA125. A total of 207 CRSs (55.1%) had both preoperative and postoperative measurements of CEA, 168 (44.7%) of CA19-9, and 171 (45.5%) of CA125 (Table). GCA cohort information is provided in eTable 1 in Supplement 1.

### Association of Complete CRS and Histology Grade With Outcomes

Historically, complete CRS and low-grade histology are associated with better OS.^7,20,33^ Similarly, complete (vs incomplete) CRS in our cohort was associated with significantly better OS (hazard ratio [HR], 0.19; 95% CI, 0.11-0.32; *P* < .001) (eFigure 2 in Supplement 1). The 5-year OS after complete CRS (86.5% [95% CI, 81.6%-91.6%]) was also greater than for incomplete CRS (45.9% [95% CI, 31.9%-66.2%]) (Table). High-grade disease was associated with significantly worse OS (HR, 4.12; 95% CI, 2.02-8.38; *P* < .001) but not with worse DFS (HR, 1.42; 95% CI, 0.98-2.06; *P* = .06) (eFigure 2 in Supplement 1).

### Association of Tumor Grade and Histology With TM Elevation

**Figure 1.**
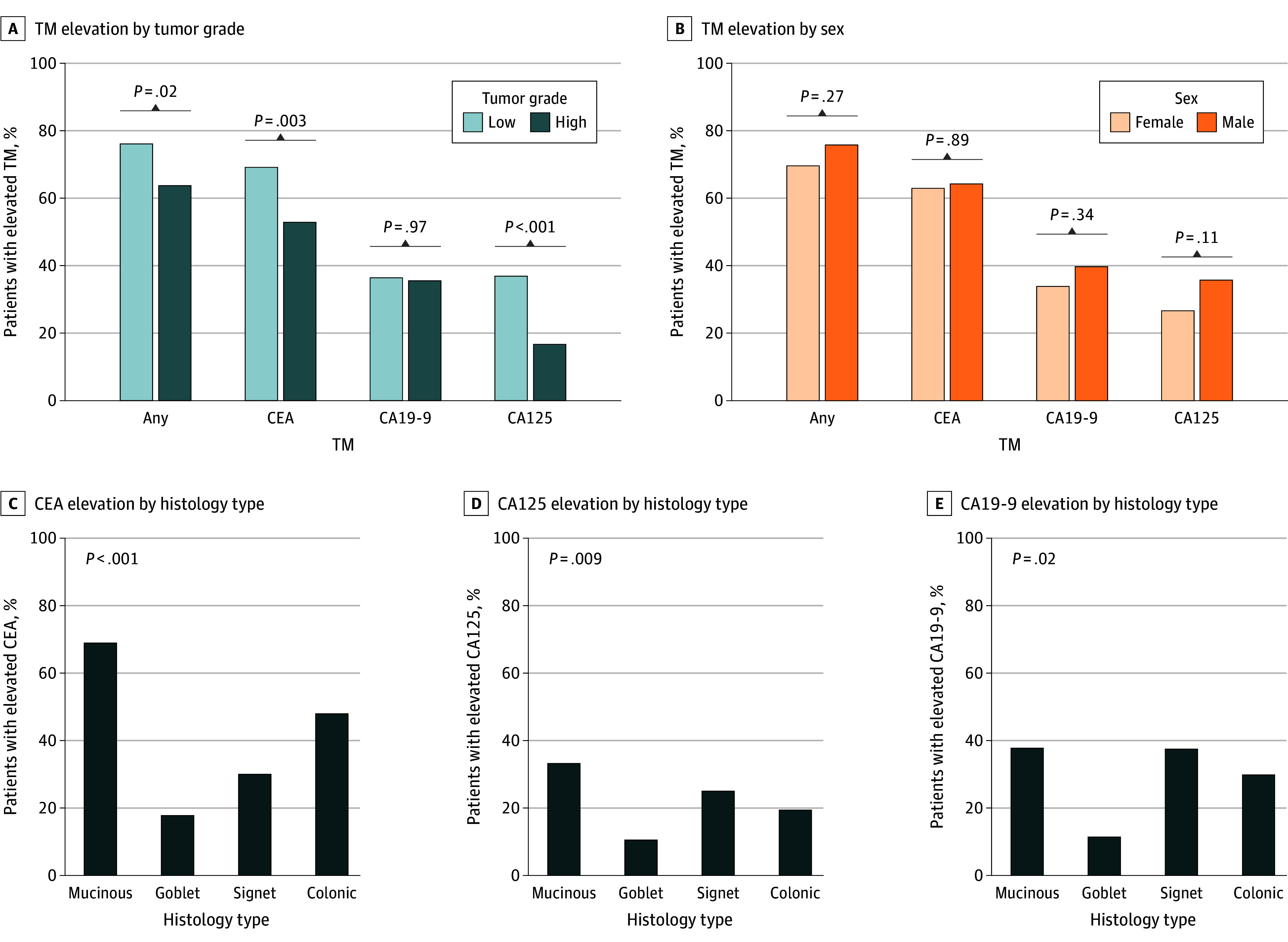
Bar Graphs Showing Preoperative Serum Tumor Marker (TM) Elevation by Sex, Tumor Grade, and Tumor Histology A, Low grade indicates well or well to moderately differentiated and high grade indicates moderately, moderately to poorly, or poorly differentiated. All *P* values are from the χ^2^ test. CA125 indicates cancer antigen 125; CA19-9, carbohydrate antigen 19-9; CEA, carcinoembryonic antigen.

We looked at various factors and their association with TM elevation. Sex had no association with elevated TMs (Figure 1B and eFigure 3 in Supplement 1). Compared with histologically high-grade tumors, low-grade tumors had significantly higher prevalence of elevated CEA and CA125 but not CA19-9 levels: CEA was elevated in 157 of 227 low-grade tumors (69.2%) vs 65 of 123 high-grade (52.8%) (*P* = .003), CA125 in 80 of 217 low-grade tumors (36.9%) vs 18 of 108 high-grade (16.7%) (*P* < .001), and CA19-9 in 79 of 217 low-grade tumors (36.4%) vs 38 of 107 high-grade (35.5%) (*P* = .97) (Figure 1A). These patterns were similar when analyzed separately in cases of complete and incomplete CRS (eFigure 3 in Supplement 1). We found that tumor histology was associated with TM levels, with mucinous tumors being associated with the most-elevated TMs and goblet cell adenocarcinomas being associated with the least (Figure 1C-E); as before, the trend was similar when analyzed separately in cases of complete and incomplete CRS (eFigure 3 in Supplement 1).

### Association of Increased Tumor Burden With Higher TM Levels

**Figure 2.**
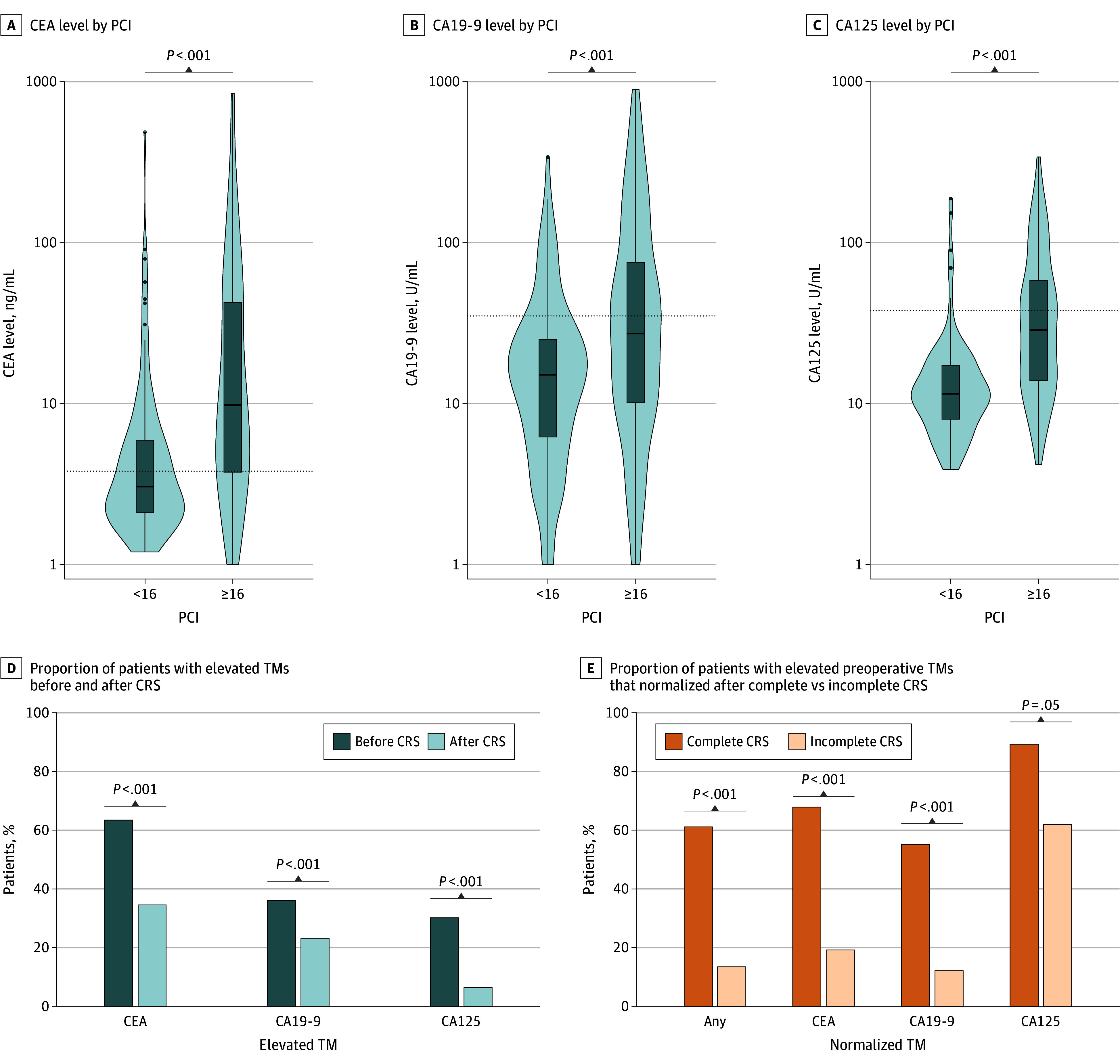
Violin Plots of Preoperative Serum Tumor Marker (TM) Levels by Appendiceal Adenocarcinoma Tumor Burden and Bar Graphs Showing Proportions of Patients With Decreased TM Elevation by Cytoreductive Surgery (CRS) Status A-C, Tumor burden was assessed using the peritoneal cancer index (PCI), stratified as low (<16) or high (≥16) on a scale from 0 to 39, with higher values indicating increased peritoneal disease burden; the y-axes are truncated at 1000, dashed horizontal lines indicate the laboratory upper limit of normal, and *P* values are from the Wilcoxon signed-rank test. The horizontal bar inside the boxes indicates the median and the lower and upper ends of the boxes, the first and third quartiles; whiskers indicate 1.5 × IQR, and shading indicates kernal density estimation. B and C, *P* values are from the χ^2^ test. CA125 indicates cancer antigen 125; CA19-9, carbohydrate antigen 19-9; CEA, carcinoembryonic antigen.

We studied the association of PCI with TM levels. Higher PCI (≥16) vs lower PCI (<16) was associated with significantly greater median levels of all 3 TMs (CEA: 3.0 ng/mL [IQR, 2.1-5.9 ng/mL] for low PCI vs 9.5 ng/mL [IQR, 3.7-42.3 ng/mL] for high [*P* < .001]; CA19-9: 15.0 U/mL [IQR, 6.1-25.5 U/mL] for low vs 31.1 U/mL [IQR, 10.0-102.3 U/mL] for high [*P* < .001]; CA125: 11.5 U/mL [IQR, 8.0-17.3 U/mL] for low vs 28.7 U/mL [IQR, 13.9-58.5 U/mL] for high [*P* < .001]) (Figure 2A-C). The trend was similar when analyzed separately in cases of complete and incomplete CRS (eFigure 4 in Supplement 1). In all, 173 of 238 low-grade tumors (72.7%) had a PCI of 16 or higher, while only 62 of 128 high-grade tumors (48.4%) did. The percentage of patients with TM elevation decreased following CRS (CEA: 222 of 340 [65.3%] before vs 77 of 223 [34.5%] after [*P* < .001]; CA19-9: 117 of 324 [36.1%] before vs 45 of 194 [23.2%] after [*P* < .001]; CA125: 98 of 325 [30.2%] before vs 13 of 203 [6.4%] after [*P* < .001]) (Figure 2D). The trend was similar when analyzed separately in cases of complete and incomplete CRS (eFigure 4 in Supplement 1). Among patients with GCA, higher PCI was not associated with significantly greater levels of TMs, and only the percentage of patients with elevated CA19-9 was lower following CRS (4 of 35 [11.4%] before vs 1 of 25 [4.0%] after; *P* < .001) (eFigure 5 in Supplement 1).

Among patients with paired preoperative and postoperative TM measurements, most had a decrease in TM measurements following either complete or incomplete CRS (eFigure 4 in Supplement 1). However, complete CRS was associated with normalized TM levels in a greater percentage of patients than was incomplete CRS (any normalized TM, 55 of 90 [61.1%] for complete vs 7 of 52 [13.5%] for incomplete CRS [*P* < .001]; normalized CEA, 57 of 84 [67.9%] vs 10 of 52 [19.2%] [*P* < .001]; normalized CA19-9, 16 of 29 [55.2%] vs 4 of 33 [12.1%] [*P* < .001]; normalized CA125, 25 of 28 [89.3%] vs 13 of 21 [61.9%] [*P* = .050]) (Figure 2E).

### Association of Preoperative TM Measurements With Completeness of CRS and With DFS

**Figure 3.**
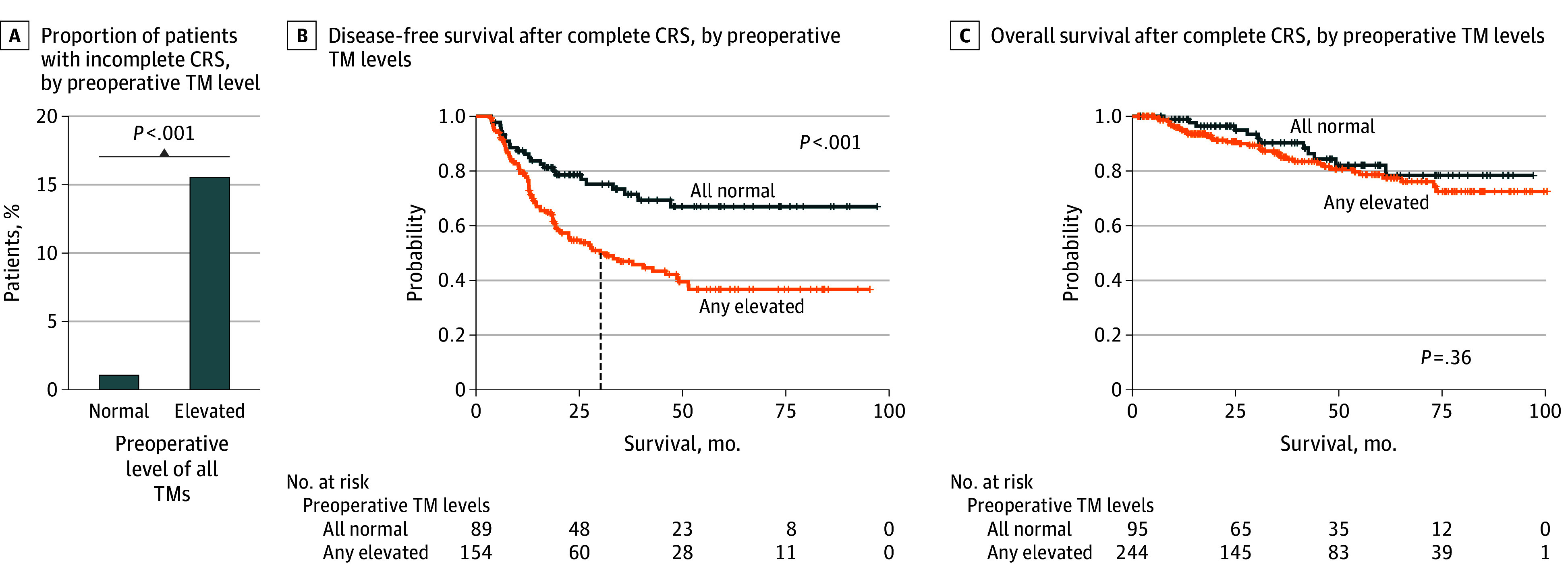
Bar Graph of Cytoreductive Surgery (CRS) Status by Preoperative Tumor Marker Measurements and Kaplan-Meier Curves Showing Survival After Complete CRS by Preoperative Serum Tumor Marker (TM) Levels A, *P* value is from the χ^2^ test. B and C, *P* values are from Cox proportional hazards regression models; dashed vertical line indicates median survival.

Of 321 curative-intent CRSs in patients with AA (85.4% of all CRSs), 33 (10.3%) were incomplete. The rate of incomplete CRS was higher in patients undergoing curative-intent CRS with preoperative TM elevation than in patients with normal preoperative TM levels (1 of 95 [1.1%] in patients with all TMs normal vs 30 of 193 [15.5%] in patients with any TM elevated; *P* < .001) (Figure 3A). These differences in rates of incomplete CRS remained when each individual TM was evaluated (3 of 124 [2.4%] with normal vs 27 of 175 [15.4%] with elevated preoperative CEA [*P* < .001]; 11 of 198 [5.6%] with normal vs 19 of 81 [23.5%] with elevated CA19-9 [*P* < .001]; 14 of 203 [6.9%] with normal vs 15 of 74 [20.3%] with elevated CA125 [*P* = .003]) (eFigure 6 in Supplement 1).

After complete CRS, patients with preoperative elevation of any TMs had worse DFS than those with normal preoperative levels of all TMs (HR, 2.30; 95% CI, 1.46-3.64; *P* < .001) (Figure 3B). The 5-year DFS in patients with any preoperative TM elevated was also worse than in those with normal preoperative TM levels (any elevated: 36.7% [95% CI, 28.4%-47.3%]; all normal: 67.0% [95% CI, 56.4%-79.5%]). When split into individual TMs, preoperative CEA and CA19-9 elevation were associated with worse DFS, while preoperative CA125 elevation was not (CEA: HR, 1.80; 95% CI, 1.22-2.67; *P* = .003; CA19-9: HR, 2.04; 95% CI, 1.34-3.09; *P* < .001; CA125: HR, 1.30; 95% CI, 0.84-2.02; *P* = .23) (eFigure 6 in Supplement 1). When TMs were analyzed collectively, preoperative elevation of any TM was not associated with worse OS (HR, 1.36; 95% CI, 0.71-2.60; *P* = .36) (Figure 3C and eTable 2 in Supplement 1). However, preoperative CA19-9 elevation by itself was associated with worse OS (HR, 2.51; 95% CI, 1.42-4.42; *P* = .002) (eFigure 7 in Supplement 1). Survival analyses of DFS in patients with GCA were limited by sample size (preoperative TM elevation: HR, 0.73; 95% CI, 0.09-6.18; *P* = .77; postoperative: HR, 1.65; 95% CI, 0.39-6.95; *P* = .50) (eFigure 8 in Supplement 1).

### Association of Postoperative TM Measurements and TM Normalization With DFS and OS

**Figure 4.**
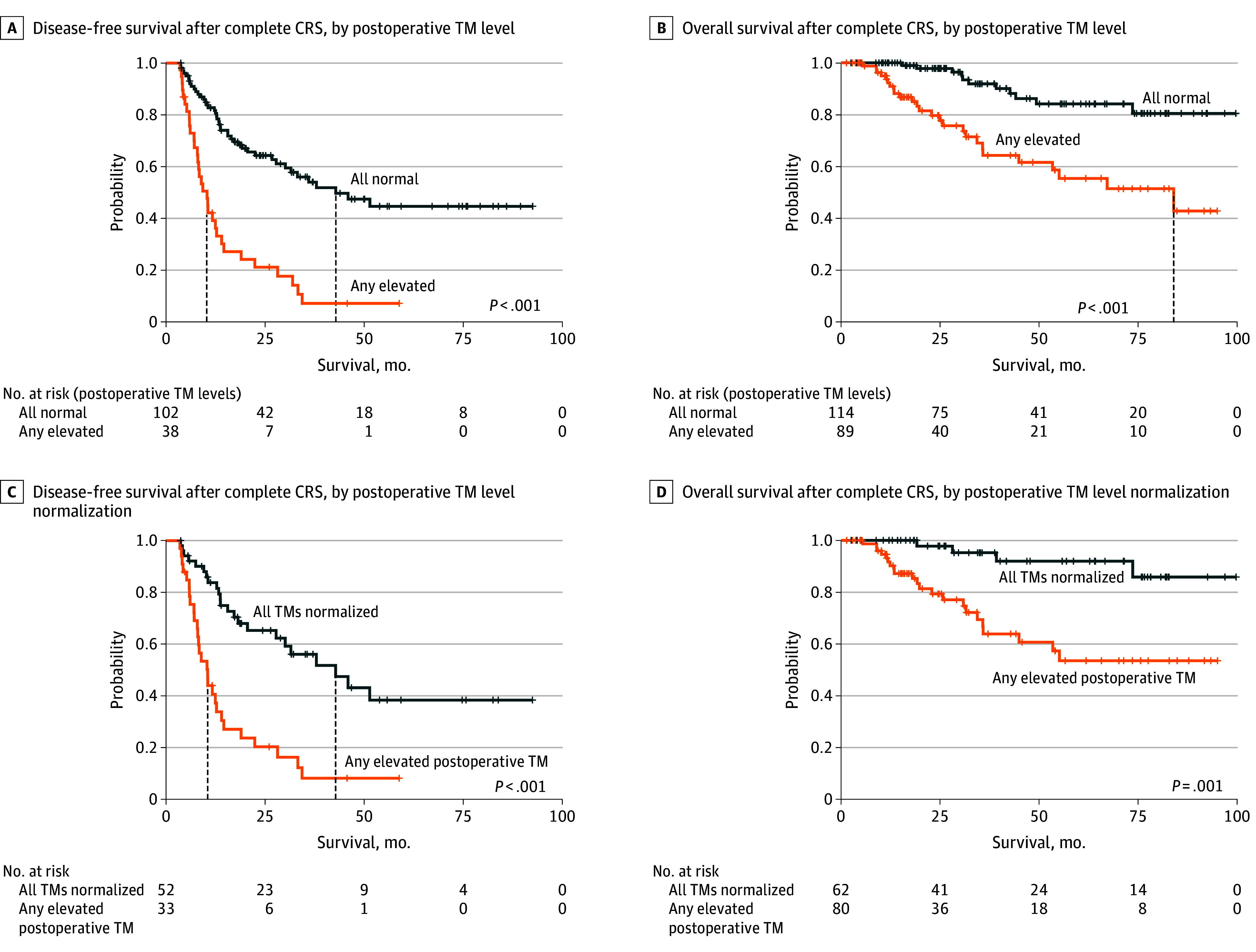
Kaplan-Meier Curves Showing Survival Within 180 Days After Cytoreductive Surgery (CRS) by Serum Tumor Marker (TM) Measurements and Normalization Dashed vertical lines indicate median survival. *P* values are from Cox proportional hazards regression models.

Having any TM elevated postoperatively (vs normal level) was associated with significantly shorter DFS (HR, 3.73; 95% CI, 2.33-5.95; *P* < .001) and lower 5-year DFS (0.0% [95% CI, 0.0%-25.8%] vs 44.5% [95% CI, 33.6%-59.1%]) (Figure 4A). Similarly, having any TM elevated postoperatively (vs normal level) was associated with worse OS (HR, 4.10; 95% CI, 2.02-8.31; *P* < .001) and lower 5-year survival (55.5% [95% CI, 42.7%-72.1%] vs 84.2% [95% CI, 75.4%-94.0%]) (Figure 4B). This association was robust to the removal of palliative CRS (HR, 3.00; 95% CI, 1.36-6.63; *P* = .006) (eTable 2 in Supplement 1) and was similar when conducted separately for complete and incomplete CRS (eFigure 9 in Supplement 1). Having any TM elevated postoperatively continued to be associated with worse OS after controlling for CRS completeness, PCI, tumor grade, and mucinous histology in a multivariate Cox proportional hazards regression model (HR, 3.34; 95% CI, 1.44-7.75; *P* = .005) (eFigure 14 in Supplement 1). Individually, postoperative elevation of CEA and CA19-9 (vs normal levels) was associated with worse DFS, while postoperative CA125 elevation was not (CEA: HR, 3.96; 95% CI, 2.45-6.40; *P* < .001; CA19-9: HR, 2.64; 95% CI, 1.35-5.18; *P* = .005; CA125: HR, 1.07; 95% CI, 0.26-4.39; *P* = .92) (eFigure 10 in Supplement 1). Postoperative elevation of all 3 individual TMs (vs normal levels) was associated with worse OS (CEA: HR, 2.33; 95% CI, 1.27-4.27; *P* = .006; CA19-9: HR, 5.56; 95% CI, 2.82-10.97; *P* < .001; CA125: HR, 5.50; 95% CI, 2.40-12.61; *P* < .001) (eFigure 11 in Supplement 1).

In patients with elevated preoperative TM levels, postoperative normalization of all TMs (vs any elevated postoperative TM) was associated with significantly longer DFS (HR, 3.54; 95% CI, 2.01-6.23; *P* < .001) and superior 5-year DFS (any elevated postoperative TM: 0.0% [95% CI, 0.0%-29.3%]; all TMs normalized: 38.3% [95% CI, 24.0%-61.1%]) (Figure 4C). When split into individual TMs, CEA normalization was significantly associated with longer DFS, while CA19-9 and CA125 normalization were not (CEA: HR, 4.16; 95% CI, 2.27-7.64; *P* < .001; CA19-9: HR, 2.17; 95% CI, 0.87-5.41; *P* = .10; CA125: HR, 0.77; 95% CI, 0.10-5.96; *P* = .81) (eFigure 12 in Supplement 1). Analysis of OS in patients with GCA was limited by sample size but showed no worse OS for preoperative (HR, 3.71; 95% CI, 0.97-15.79; *P* = .08) or postoperative (HR, 1.96; 95% CI, 0.52-7.35; *P* = .32) TM elevation vs normal levels (eFigure 8 in Supplement 1).

Postoperative normalization of all TMs (vs any elevated postoperative TM) was also associated with improved OS (HR, 6.00; 95% CI, 2.06-17.46; *P* = .001) and greater 5-year survival (elevated level of any TM: 53.4% [95% CI, 39.7%-71.9%]; all TMs normalized: 91.9% [95% CI, 83.4%-100.0%]) (Figure 4D). This association was robust to the removal of palliative CRS (HR, 3.92; 95% CI, 1.23-12.55; *P* = .02) (eTable 2 in Supplement 1). TM normalization was not associated with better OS after complete CRS (HR, 2.64; 95% CI, 0.74-9.43; *P* = .13); after incomplete CRS, there were no observed events with all TMs normalized (eFigure 13 in Supplement 1). TM normalization continued to be associated with improved OS after controlling for CRS completeness, PCI, grade, and mucinous histology in a multivariate Cox proportional hazards regression model (HR, 4.21; 95% CI, 1.33-13.35; *P* = .02) (eFigure 14 in Supplement 1). Normalization of CEA and CA125 was associated with improved OS (CEA: HR, 3.90; 95% CI, 1.53-9.96; *P* = .004; CA125: HR, 7.16; 95% CI, 1.83-28.02; *P* = .005); no events were observed with normalized CA19-9 (eFigure 12 in Supplement 1).

## Discussion

This study reports results of a large, retrospective cohort analysis evaluating the association between preoperative and postoperative levels of CEA, CA19-9, and CA125 in patients undergoing CRS with or without HIPEC for AA with peritoneal dissemination. It adds to the growing literature regarding how TMs can be used to manage the care of patients with metastatic AA.^19^

We found that preoperative TM levels were associated with AA tumor burden as measured by operative assessment of PCI. When tumor burden was decreased by CRS, there was a concomitant decrease in CEA, CA19-9, and CA125 levels. As expected, complete CRS was more likely to be associated with normalized CEA, CA19-9, and CA125 compared with incomplete CRS, consistent with serum TM levels being an accurate surrogate marker of tumor burden. Notably, in curative-intent CRS, incomplete CRS was uncommon, occurring only 10.3% of the time. However, in curative-intent CRS, there was only a 1.1% risk of incomplete CRS if all 3 TMs were normal preoperatively, while if any TM was elevated, there was a 15.5% risk of incomplete CRS. This represents a 14-fold greater risk of incomplete CRS with elevated TMs. Since it can be difficult to assess disease burden and other disease features in the peritoneal cavity using traditional cross-sectional imaging,^34^ this finding suggests that preoperative TM measurement could help identify patients at risk for incomplete CRS. Additionally, when restricting to complete CRS, elevation of TM preoperatively was associated with shorter DFS. This suggests that TM elevation may also be a surrogate marker of more aggressive tumor biology. Furthermore, there are data from preclinical models suggesting that in addition to being tumor markers, both CEA and CA19-9 may promote metastasis, immune evasion, and/or angiogenesis and tumor proliferation rate.^35,36^ Similarly, CA125 is known to promote tumorigenesis, invasion, and metastasis,^37^ potentially by activating the Wnt pathway.^38^

We found that higher histologic grade was associated with a decreased proportion of patients with elevated TMs; however, previous research found that histologic grade was not associated with TM levels.^19^ We interpret this as being due to patient selection: low-grade tumors are often operated on regardless of PCI and location of metastatic deposits, while high-grade tumors are typically operated on only if a complete CRS can be achieved.^2^ As a result, high-grade tumors that are operated on typically have lower PCI and lower TM levels. As expected, 72.7% of low-grade AA tumors in our CRS cohort had PCI of 16 or greater, while only 48.4% of high-grade tumors did.

We also found an association between TM elevation in the 180-day postoperative period and worse DFS and OS. Elevation of any TM postoperatively was associated with more than 3-fold worse DFS. Of interest, no patients with complete CRS were still disease-free after 5 years if they had any postoperative TM elevation, vs 44.5% of patients when all postoperative TMs were normal. This result suggests that more frequent surveillance may be needed in the very high-risk population with postoperative TM elevation. Patients in whom preoperative TM elevation was not normalized after CRS represented another high-risk cohort. None of these patients remained disease-free after 5 years, vs 38.3% when all TMs were normalized after complete CRS. Notably, CRS without TM normalization was associated with a nearly 6-fold increased risk of death within 5 years compared with CRS in patients with normalized TMs, an association that was robust to controlling for clinicopathologic features associated with worse OS. In addition, the HR for TM normalization was higher than for postoperative TM elevation alone, suggesting that both preoperative and postoperative TMs are necessary to optimally risk stratify patients undergoing CRS.

In recent years, it has become clear that AA is a distinct molecular and clinical entity from colorectal cancer.^34,39,40^ Despite this, AA is often treated the same as colorectal cancer due to a lack of clinical trials specifically studying AA.^41^ Since it is hard to measure peritoneal metastases using traditional Response Evaluation Criteria in Solid Tumors criteria, progression-free survival (PFS) measurement has been challenging in AA-specific clinical trials. Not having PFS as a surrogate end point^42,43^ is problematic in AA, because given its often indolent growth rate, 10 years of follow-up would be needed to accurately assess OS.^4^ The data presented herein suggest that serial TM measurement could be used as a surrogate marker for OS, supplementing either radiographic response or PFS.^44^

### Limitations

This study has limitations. Because it was a single-institution retrospective study at a quaternary referral cancer center, our findings may be affected by referral bias. Another limitation of this study is that although pathologic diagnosis was determined by pathologists with specific expertise in appendix cancer, a central pathologic review by a single pathologist was not performed specifically for this study. Additionally, the number of patients with elevated postoperative TM levels may be too small to draw conclusions for certain groups (eg, those with elevated postoperative CA125). It should also be noted that our conclusion that TMs may be a good marker of tumor burden (and therefore treatment response) may be true only in response to CRS and not in response to systemic chemotherapies. Although TMs have been used to track systemic chemotherapy response in patients with AA,^34,40,45^ more research is needed to show the relationship between tumor response to chemotherapies and TM levels. In addition, postoperative follow-up at MDACC may have been incomplete due to geographic limitations. Many patients may have followed up at institutions closer to them, and we did not have access to postoperative data from these external institutions.

## Conclusions

This cohort study found that elevated serum levels of CEA, CA19-9, and CA125 both before and after CRS for AA were associated with worse DFS and OS. Based on the findings, we suggest that tests of all 3 TM levels be ordered at regular intervals in patients with AA. Following levels of CEA, CA19-9, and CA125 may play an important role in determining treatment response as well as preoperative and postoperative prognostication in patients undergoing CRS.

## Notes

Supplement 1. **eTable 1.** GCA Cohort Information **eTable 2.** Univariate Cox Proportional Hazards Models in Only Curative Intent Cytoreductive Surgeries **eFigure 1.** Cohort Diagram **eFigure 2.** Features Associated With Survival in Cytoreductive Surgeries in the Cohort **eFigure 3.** Effect of Sex, Grade, and Histology on Preoperative Tumor Marker Elevation in Complete and Incomplete Cytoreductive Surgery **eFigure 4.** TM Levels Are Associated With Appendiceal Adenocarcinoma Tumor Burden in Both Complete and Incomplete CRS **eFigure 5.** Relationship Between TM Levels and Tumor Burden in Patients With GCA **eFigure 6.** Preoperative Tumor Marker Measurements Separately Are Predictive of Completeness of Cytoreduction and DFS **eFigure 7.** Kaplan-Meier Plots of Overall Survival Based on Preoperative Tumor Marker Levels in Complete and Incomplete CRS **eFigure 8.** Kaplan-Meier Plots of OS and DFS in Patients With GCA With Any TMs Elevated Either Preoperatively or Postoperatively **eFigure 9.** Kaplan-Meier Plots of OS in Patients With Any TM Elevated Postoperatively **eFigure 10.** Disease-Free Survival Based on Postoperative Tumor Marker Elevation **eFigure 11.** OS Based on Postoperative Tumor Marker Levels **eFigure 12.** Kaplan-Meier Plots of Disease-Free Survival and Overall Survival in Patients With Elevated TM Levels Stratified by Postoperative Normalization **eFigure 13.** Overall Survival Stratified by Postoperative TM Normalization **eFigure 14.** Multivariable Cox Proportional Hazards Regression Models With Postoperative TM Elevation and Normalization

Supplement 2.
Data Sharing Statement
