# Supplementary material for: Appendiceal Adenocarcinoma Cytoreduction Outcomes and Perioperative Serum Tumor Marker Levels
Source: JAMA Netw Open. 2026 May 4;9(5):e2610569. doi: 10.1001/jamanetworkopen.2026.10569 (PMC13139952; doi:10.1001/jamanetworkopen.2026.10569)
Supplement: Supplement 1. — eTable 1. GCA Cohort Information eTable 2. Univariate Cox Proportional Hazards Models in Only Curative Intent Cytoreductive Surgeries eFigure 1. Cohort Diagram eFigure 2. Features Associated With Survival in Cytoreductive Surgeries in the Cohort eFigure 3. Effect of Sex, Grade, and Histology on Preoperative Tumor Marker Elevation in Complete and Incomplete Cytoreductive Surgery eFigure 4. TM Levels Are Associated With Appendiceal Adenocarcinoma Tumor Burden in Both Complete and Incomplete CRS eFigure 5. Relationship Between TM Levels and Tumor Burden in Patients With GCA eFigure 6. Preoperative Tumor Marker Measurements Separately Are Predictive of Completeness of Cytoreduction and DFS eFigure 7. Kaplan-Meier Plots of Overall Survival Based on Preoperative Tumor Marker Levels in Complete and Incomplete CRS eFigure 8. Kaplan-Meier Plots of OS and DFS in Patients With GCA With Any TMs Elevated Either Preoperatively or Postoperatively eFigure 9. Kaplan-Meier Plots of OS in Patients With Any TM Elevated Postoperatively eFigure 10. Disease-Free Survival Based on Postoperative Tumor Marker Elevation eFigure 11. OS Based on Postoperative Tumor Marker Levels eFigure 12. Kaplan-Meier Plots of Disease-Free Survival and Overall Survival in Patients With Elevated TM Levels Stratified by Postoperative Normalization eFigure 13. Overall Survival Stratified by Postoperative TM Normalization eFigure 14. Multivariable Cox Proportional Hazards Regression Models With Postoperative TM Elevation and Normalization [file jamanetwopen-e2610569-s001.pdf]

Supplementary Online Content

Pattalachinti VK, Seldomridge A, Yousef A, et al. Appendiceal adenocarcinoma cytoreduction outcomes and perioperative serum tumor marker levels. *JAMA Netw Open*. 2026;9(5):e2610569. doi:10.1001/jamanetworkopen.2026.10569

eTable 1. GCA Cohort Information

eTable 2. Univariate Cox Proportional Hazards Models in Only Curative Intent Cytoreductive Surgeries

eFigure 1. Cohort Diagram

eFigure 2. Features Associated With Survival in Cytoreductive Surgeries in the Cohort

eFigure 3. Effect of Sex, Grade, and Histology on Preoperative Tumor Marker Elevation in Complete and Incomplete Cytoreductive Surgery

eFigure 4. TM Levels Are Associated With Appendiceal Adenocarcinoma Tumor Burden in Both Complete and Incomplete CRS

eFigure 5. Relationship Between TM Levels and Tumor Burden in Patients With GCA

eFigure 6. Preoperative Tumor Marker Measurements Separately Are Predictive of Completeness of Cytoreduction and DFS

eFigure 7. Kaplan-Meier Plots of Overall Survival Based on Preoperative Tumor Marker Levels in Complete and Incomplete CRS

eFigure 8. Kaplan-Meier Plots of OS and DFS in Patients With GCA With Any TMs Elevated Either Preoperatively or Postoperatively

eFigure 9. Kaplan-Meier Plots of OS in Patients With Any TM Elevated Postoperatively

eFigure 10. Disease-Free Survival Based on Postoperative Tumor Marker Elevation

eFigure 11. OS Based on Postoperative Tumor Marker Levels

eFigure 12. Kaplan-Meier Plots of Disease-Free Survival and Overall Survival in Patients With Elevated TM Levels Stratified by Postoperative Normalization

eFigure 13. Overall Survival Stratified by Postoperative TM Normalization

eFigure 14. Multivariable Cox Proportional Hazards Regression Models With Postoperative TM Elevation and Normalization

This supplementary material has been provided by the authors to give readers additional information about their work.

© 2026 Pattalachinti VK et al. *JAMA Network Open*.

	Complete (n = 35 CRS; 77.8%)	Incomplete (n = 10; 22.2%)	Total (n = 45)
**Number of Patients (n, [% of total patients])**	33 (78.6)	10 (23.8)	42
**Age at Diagnosis (years, median [IQR])**	62 [53.5-67.5]	59 [55.5-61.75]	60 [55-67]
**Age at Surgery (years, median [IQR])**	62 [53.5-67.5]	61 [58-65.25]	61 [56-67]
**Time From Diagnosis to Surgery**			
<6 mo (n, %)	3 (8.6)	0 (0.0)	3 (6.7)
6 mo-2 yrs (n, %)	26 (74.3)	6 (60.0)	32 (71.1)
>2 yrs (n, %)	6 (17.1)	4 (40.0)	10 (22.2)
**Female Sex (n, [%])**	23 (65.7)	4 (40.0)	27 (60.0)
**Overall Survival after CRS**			
Median Survival (months [95% CI])	NR	19.3 [18.8-NR]	33.9 [28.2-NR]
5 Year Survival (% [95% CI])	50.4 [33.3-76.4]	0 [NA]	46.4 [30.2-71.0]
**Disease Free Survival (DFS)**			
Median DFS (months [95% CI])	37.7 [22.9-NR]	NA	NA
5 Year DFS (% [95% CI])	31.5 [14.1-70.6]	NA	NA
**Race/Ethnicity (Self-Reported)**			
African American/Black (n, %)	1 (2.9)	0 (0.0)	1 (2.2)
Asian (n, %)	2 (5.7)	0 (0.0)	2 (4.4)
Hispanic or Latino (n, %)	2 (5.7)	2 (20.0)	4 (8.9)
White or Caucasian, Non-Hispanic (n, %)	28 (80.0)	8 (80.0)	36 (80.0)
Other, unknown, or declined to answer (n, %)	2 (5.7)	0 (0.0)	2 (4.4)
**Peritoneal Cancer Index (median [IQR])**	15 [8.5-19.5]	26 [20-29]	16.5 [9-24]
**Grade**			
Well- and Well to Moderately-Differentated (n, %	3 (8.8)	0 (0.0)	3 (6.8)
Moderately- and Poorly-Differentiated (n, %)	31 (91.2)	10 (100.0)	41 (93.2)
**Microsatellite stability**			
MSS (n, %)	22 (100.0)	6 (100.0)	28 (100.0)
MSI-H (n, %)	0 (0.0)	0 (0.0)	0 (0)
**Preoperative Tumor Markers**			
CEA (n, %)	35 (100.0)	10 (100.0)	45 (100.0)
CA19-9 (n, %)	28 (80.0)	7 (70.0)	35 (77.8)
CA125 (n, %)	31 (88.6)	7 (70.0)	38 (84.4)
**Postoperative Tumor Markers**			
CEA (n, %)	28 (80.0)	8 (80.0)	36 (80.0)
CA19-9 (n, %)	20 57.1)	5 (50.0)	25 (56.6)
CA125 (n, %)	22 (62.9)	6 (60.0)	28 (62.2)
**Paired Tumor Markers**			
CEA (n, %)	28 (80.0)	8 (80.0)	36 (80.0)
CA19-9 (n, %)	18 (51.4)	2 (20.0)	20 (44.4)
CA125 (n, %)	21	3 (30.0)	24 (53.3)
**Repeat CRS**			
1st CRS (n, %)	30	9 (90.0)	39 (86.7)
2nd CRS (n, %)	5	1 (10.0)	6 (13.3)
3rd CRS (n, %)	0 (0)	0 (0.0)	0 (0.0)
**ECOG**			
0 (n, %)	22	4 (40.0)	26 (57.8)
1 (n, %)	8	5 (50.0)	13 (28.9)
2 (n, %)	0	0 (0.0)	0 (0.0)
3 (n, %)	0	0 (0.0)	0 (0.0)
**HIPEC**			
No HIPEC	4	9 (90.0)	13 (28.9)
Mitomycin C	29	1 (10.0)	30 (66.7)
Oxaliplatin	0	0 (0.0)	0 (0.0)
Cisplatin	2	0 (0.0)	2 (4.4)

Supplemental Table 1: GCA Cohort information. Abbreviations: CA125, cancer antigen 125, CA19-9 carbohydrate antigen 19-9, CEA, carcinoembryonic antigen, NR = not reached. Other race includes Native Hawaiian, Other Pacific Islander, American Indian, or Alaska Native.

© 2026 Pattalachinti VK et al. *JAMA Network Open*.

	HR (OS)	P
Any TM Elevated Preoperatively	0.89	0.76
**Any TM Elevated Postoperatively**	**3.00**	**0.006**
**TMs Normalized Postoperatively**	**3.92**	**0.02**
CEA Preop Elevated	0.64	0.18
CEA Postop Elevated	1.70	0.19
CA19-9 Preop Elevated	1.90	0.07
**CA19-9 Postop Elevated**	**5.59**	**<.001**
CA125 Preop Elevated	0.82	0.60
**CA125 Postop Elevated**	**5.96**	**0.02**

Supplemental Table 2: Univariate Cox proportional hazards models in only curative intent cytoreductive surgeries. TM = Tumor Markers, CA125, cancer antigen 125, CA19-9 carbohydrate antigen 19-9, CEA, carcinoembryonic antigen

© 2026 Pattalachinti VK et al. *JAMA Network Open*.

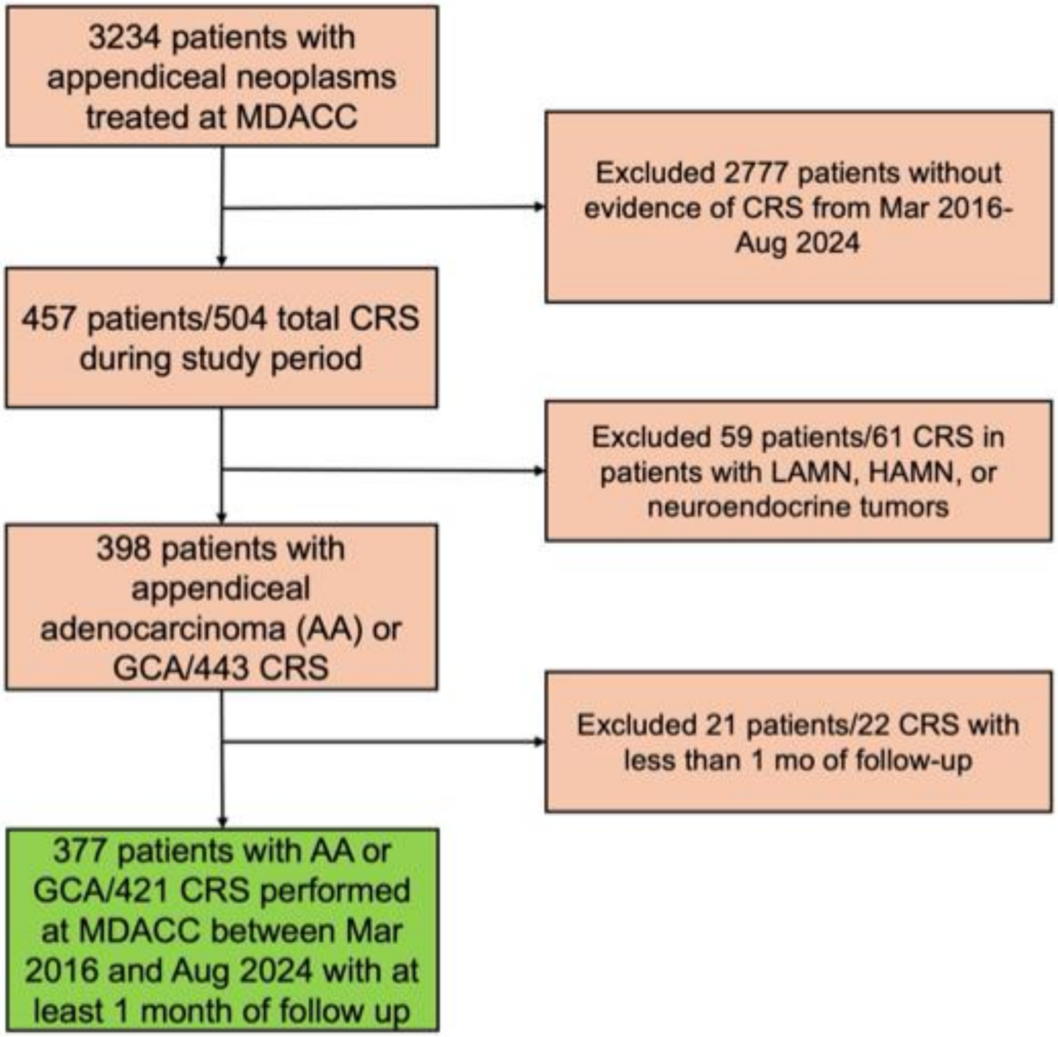

Supplemental Figure 1: Cohort Diagram

© 2026 Pattalachinti VK et al. *JAMA Network Open*.

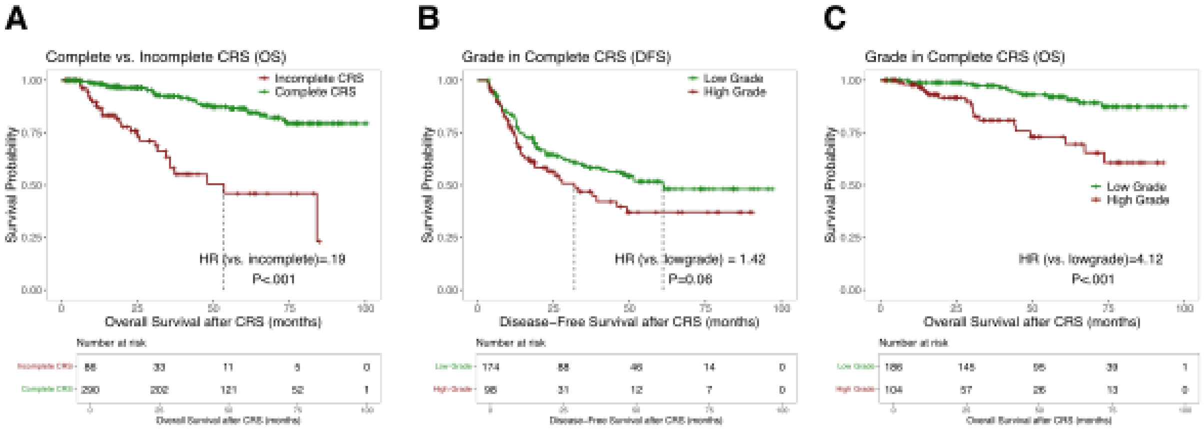

Supplemental Figure 2: Features associated with survival in cytoreductive surgeries (CRS) are also found in our cohort. **2A**: Kaplan-Meier (KM) plot of OS stratified by complete vs. incomplete CRS. **2B**: KM plot of OS stratified by low grade (well- or well-to-moderately differentiated) or high-grade (moderately, moderately-to-poor, or poorly differentiated) histology. **2C**: KM plot of disease-free survival (DFS) stratified by low-grade or high-grade histology. HR and P by Cox Proportional Hazards Model.

© 2026 Pattalachinti VK et al. *JAMA Network Open*.

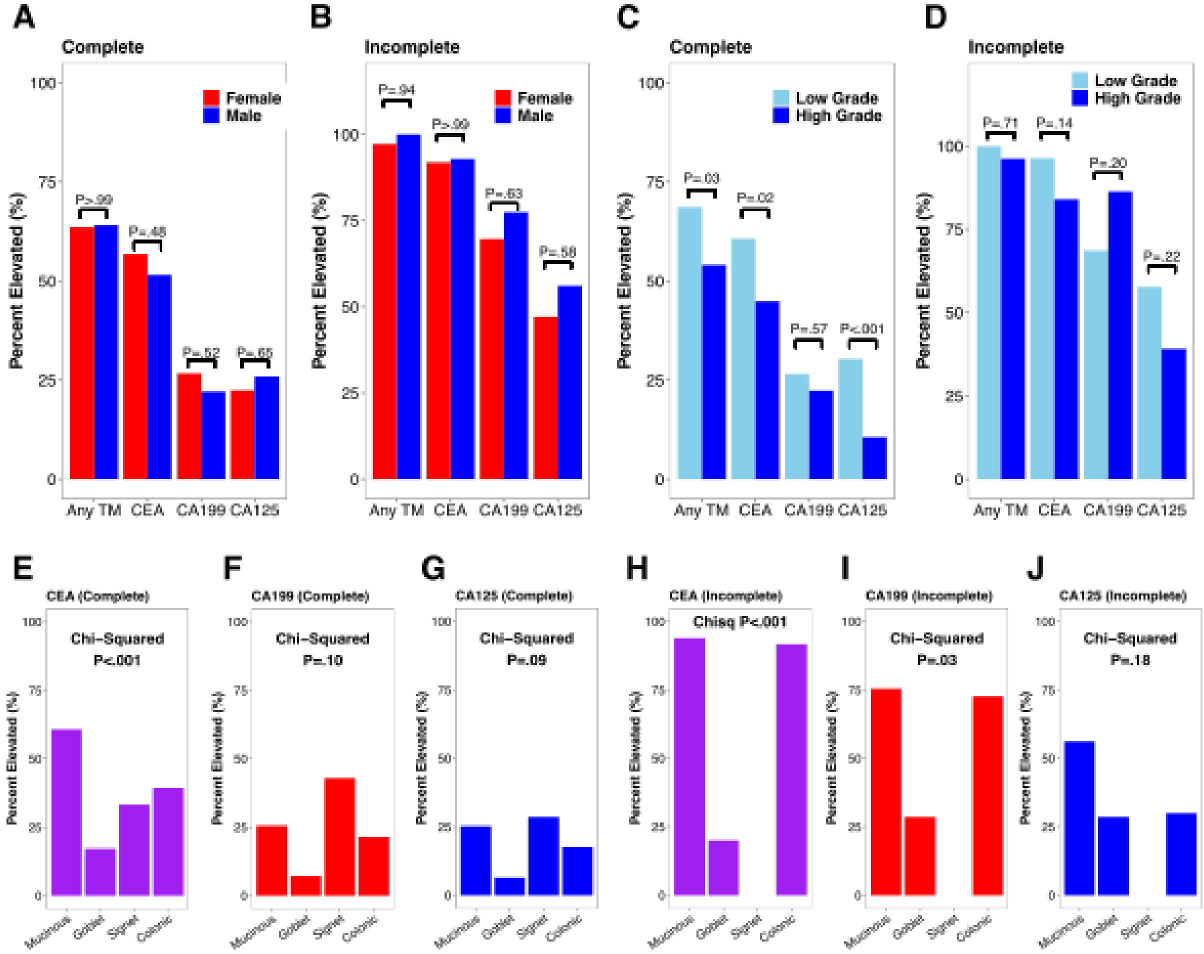

Supplemental Figure 3: Effect of sex, grade, and histology on preoperative tumor marker elevation in complete and incomplete cytoreductive surgery (CRS). **3A**: Effect of sex in complete CRS. **3B**: Effect of sex in incomplete CRS. **3C**: Effect of tumor grade in complete CRS. Low grade = well-differentiated or well- to moderately-differentiated. **3D**: Effect of tumor grade in incomplete CRS. **3E**: Effect of histology in complete CRS on CEA elevation. **3F**: Effect of histology in complete CRS on CA199 elevation. **3G**: Effect of histology incomplete CRS on CA125 elevation. **3H**: Effect of histology in incomplete CRS on CEA elevation. **3I**: Effect of histology in incomplete CRS on CA199 elevation. **3J**: Effect of histology in incomplete CRS on CA125 elevation

© 2026 Pattalachinti VK et al. *JAMA Network Open*.

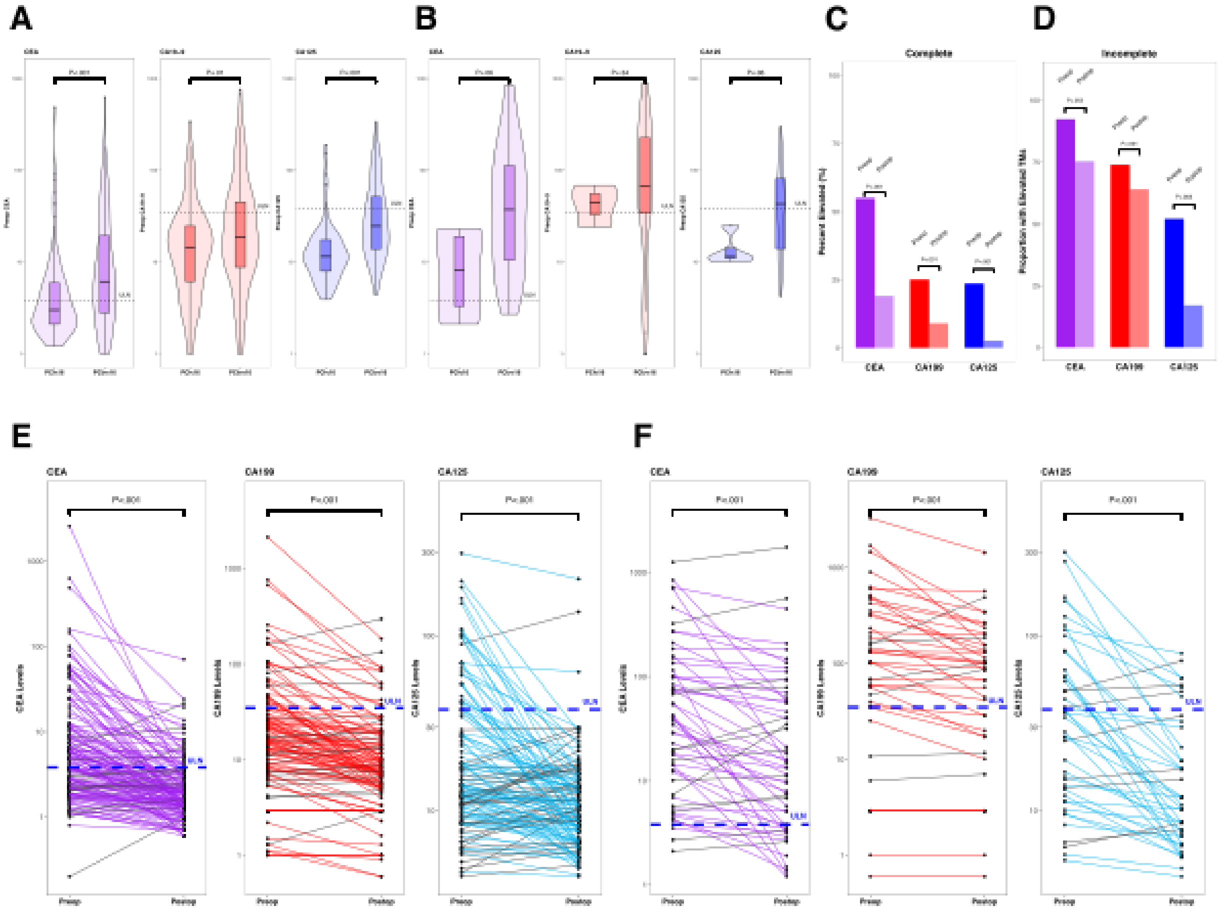

Supplemental Figure 4: TM levels are associated with appendiceal adenocarcinoma tumor burden in both complete and incomplete CRS. **4A**: Levels of preoperative tumor marker levels in patients with low (<16) and high (≥16) peritoneal carcinomatosis index (PCI) in complete CRS. Y-axis truncated at 1000. P-val by Wilcoxon. **4B**: Levels of preoperative tumor marker levels in patients with low (<16) and high (≥16) peritoneal carcinomatosis index (PCI) in incomplete CRS. Y-axis truncated at 1000. P-val by Wilcoxon. **4C**: Percent of CRS with preoperative and postoperative elevated TMs in complete CRS. P-val by chi-squared test. **4D**: Percent of CRS with preoperative and postoperative elevated TMs in incomplete CRS. P-val by chi-squared test. **4E**: Paired levels of preoperative and postoperative tumor marker levels in complete cytoreductions. P-val by paired Wilcoxon. **4F**: Paired levels of preoperative and postoperative tumor marker levels in incomplete cytoreductions. P-val by paired Wilcoxon.

© 2026 Pattalachinti VK et al. *JAMA Network Open*.

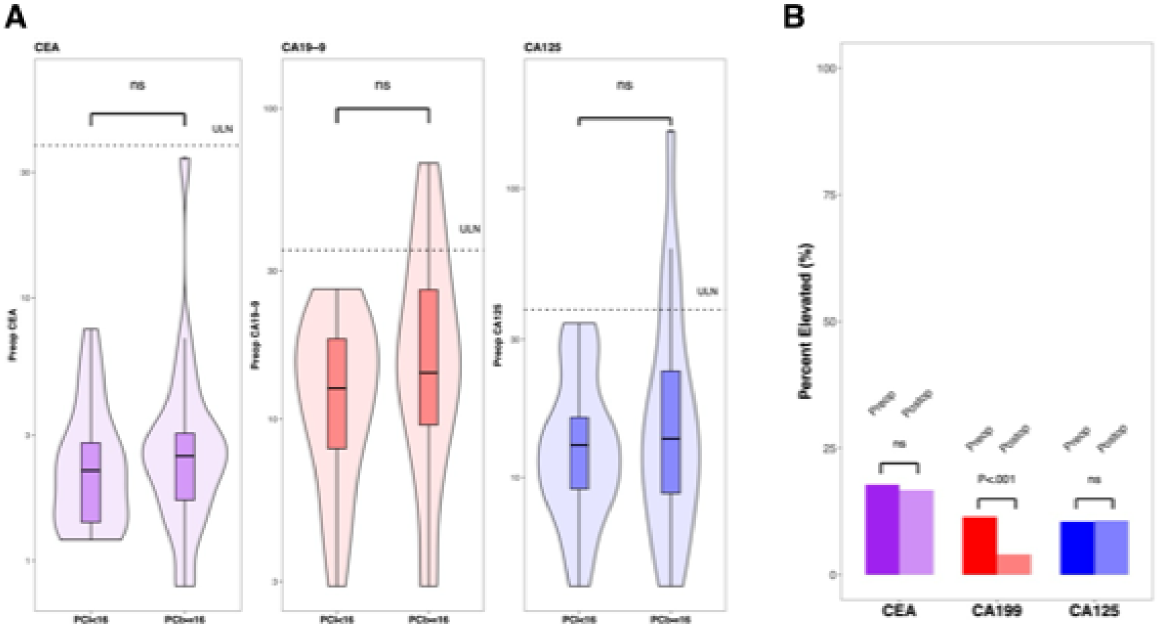

Supplemental Figure 5. Relationship between TM levels and tumor burden in patients with GCA **5A**: Levels of preoperative tumor marker levels in patients with GCA with low (<16) and high (≥16) peritoneal carcinomatosis index (PCI). Yaxis truncated at 1000. P-val by Wilcoxon. **5B**: Percent of CRS with preoperative and postoperative elevated TMs in patients with GCA. P-val by chi-squared test

© 2026 Pattalachinti VK et al. *JAMA Network Open*.

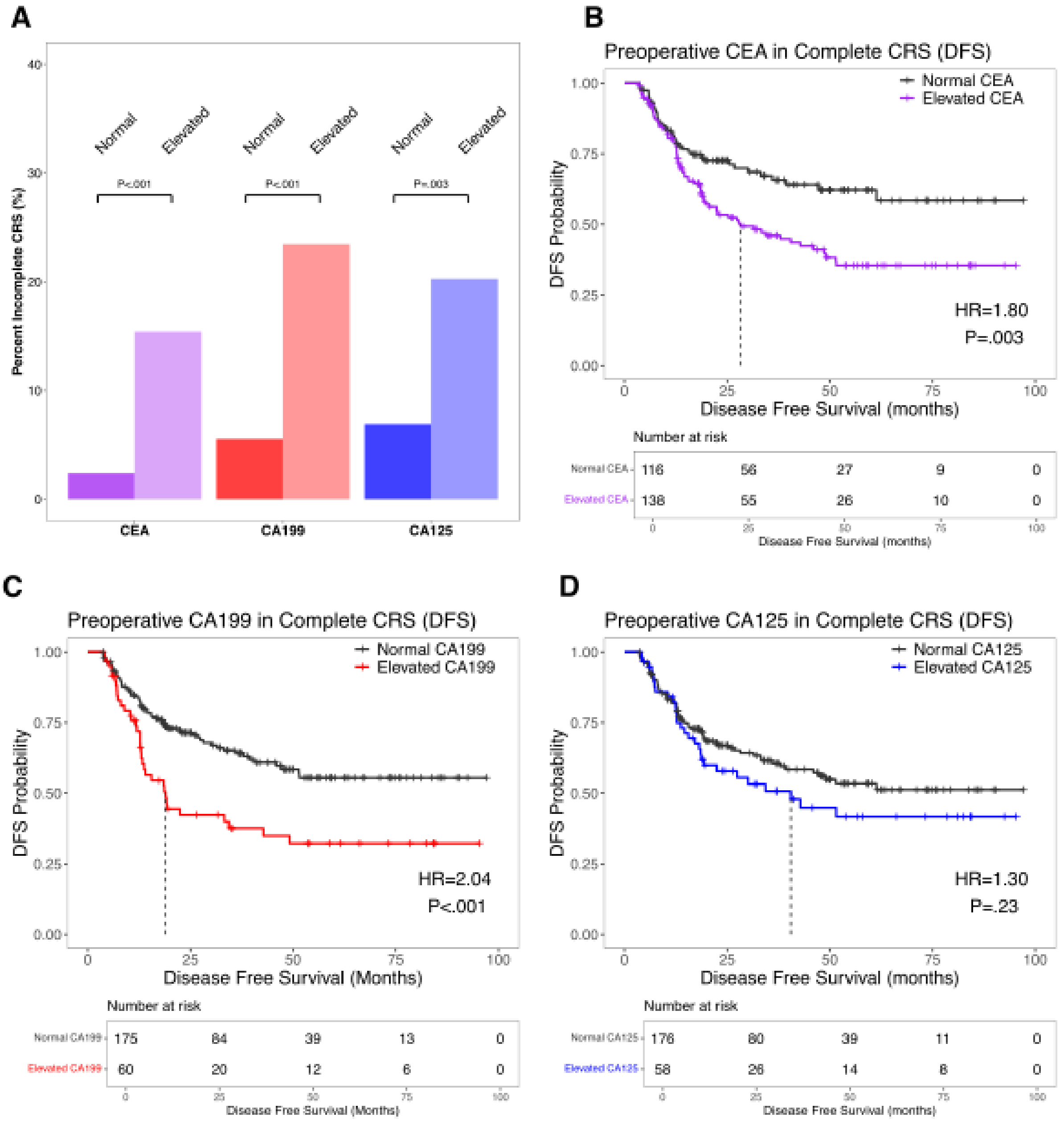

Supplemental Figure 6: Preoperative tumor marker measurements separately are predictive of completeness of cytoreduction and DFS. **6A**: Comparison of percent of incomplete cytoreductions in patients with elevated or normal preoperative tumor markers CEA, CA19-9, or CA125 who underwent curative-intent CRS. **6B**: KM plot of DFS stratified on preoperative CEA elevation. **6C**: KM plot of DFS stratified on preoperative CA199 elevation. **6D**: KM plot of DFS stratified on preoperative CA125 elevation. HR elevated vs normal. HR and P-val by Cox proportional hazards test.

© 2026 Pattalachinti VK et al. *JAMA Network Open*.

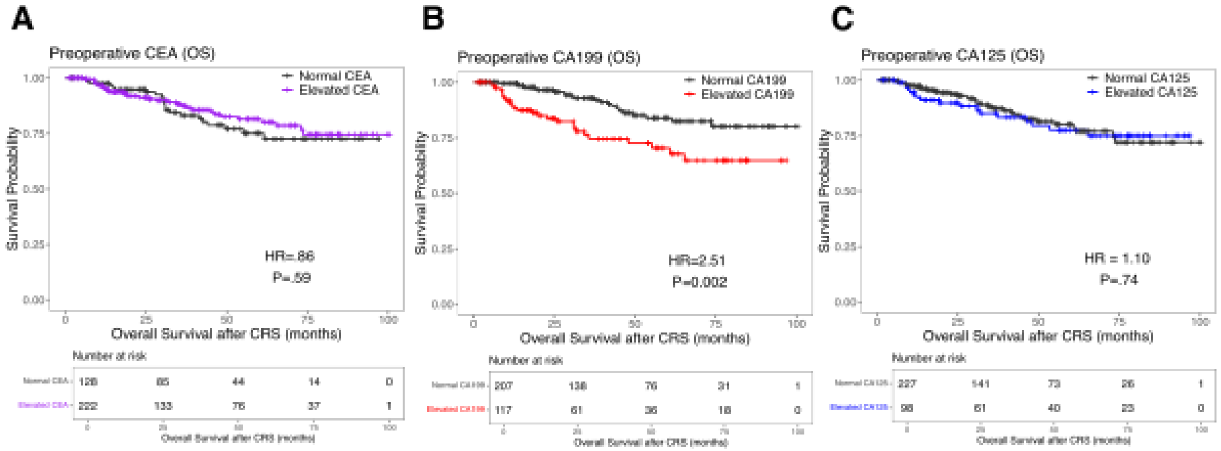

Supplemental Figure 7: Kaplan-Meier plots of overall survival (OS) based on preoperative tumor marker levels in complete and incomplete CRS. **7A**: KM plot of OS stratified on preoperative CEA elevation **7B**: KM plot of OS stratified on preoperative CA199 elevation **7C**: KM plot of OS stratified on preoperative CA125 elevation. All HR are elevated vs normal. HR and P-val by Cox proportional hazards test.

© 2026 Pattalachinti VK et al. *JAMA Network Open*.

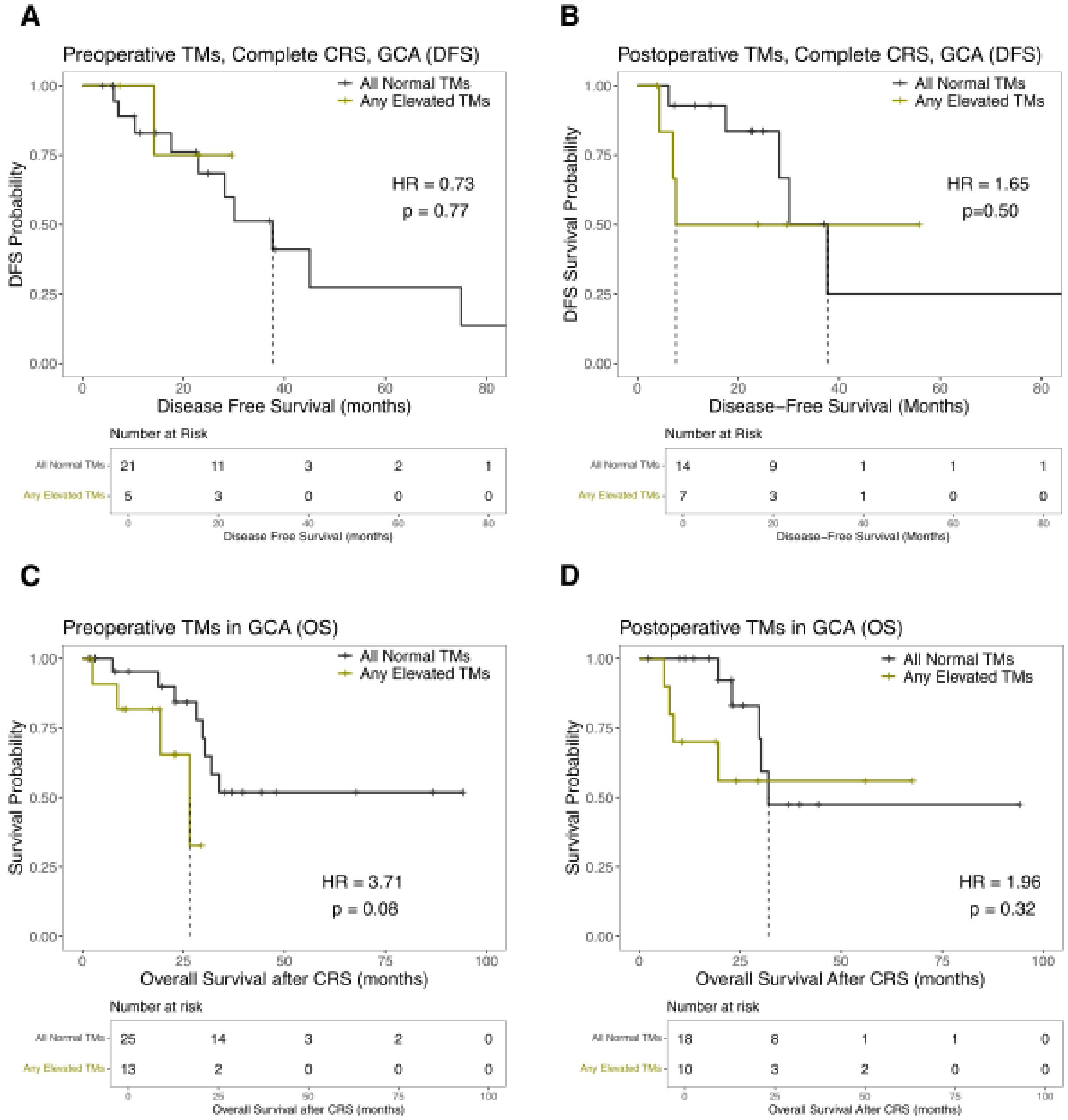

Supplemental Figure 8: Kaplan Meier Plots of OS and DFS in patients with GCA with any TMs elevated either preoperatively or postoperatively. **8A**: KM plot of DFS in patients with GCA with elevated vs. normal preoperative TMs. **8B**: KM plot of DFS in patients with GCA with elevated vs. normal postoperative TMs. **8C**: KM plot of OS in patients with GCA with elevated vs. normal preoperative TMs. **8D**: KM plot of OS in patients with GCA with elevated vs. normal postoperative TMs. HR and P-val by Cox Proportional Hazards Test.

© 2026 Pattalachinti VK et al. *JAMA Network Open*.

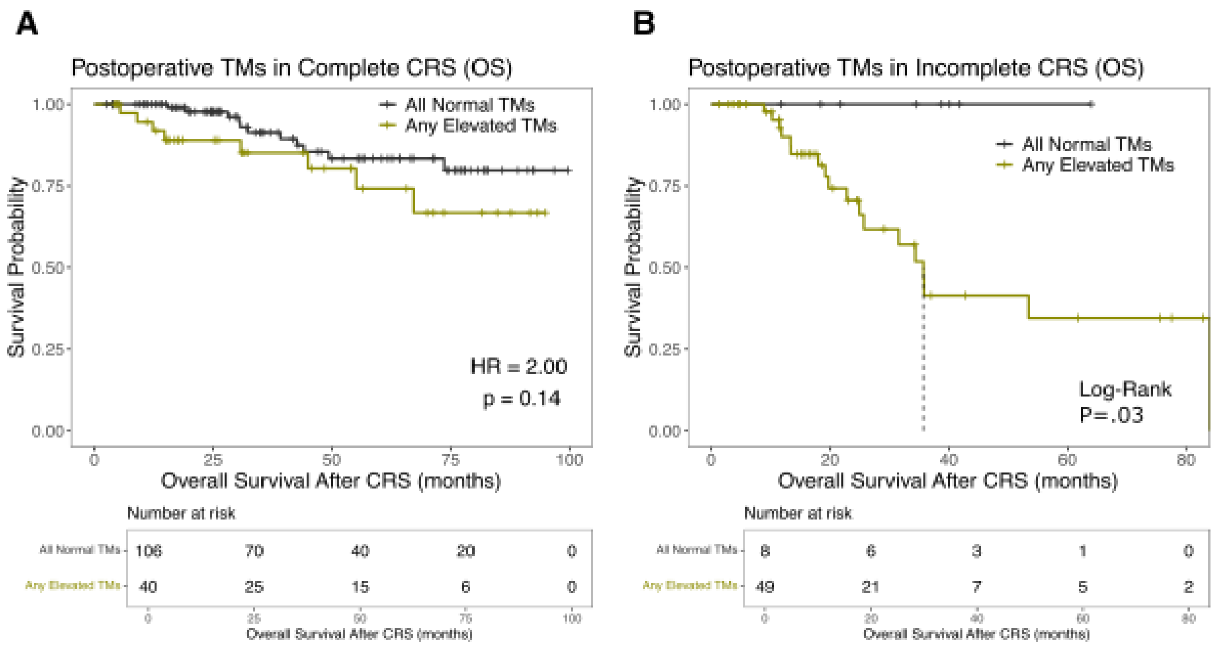

Supplemental Figure 9: Kaplan-Meier plots of OS in patients with any TM elevated postoperatively. **9A**: KM plot of OS in patients with elevated or normal postoperative TMs that underwent complete CRS. **9B**: KM plot of OS in patients that underwent incomplete CRS with elevated or normal postoperative TMs. All HR are elevated vs normal. P-val by Cox proportional hazards test or log-rank test.

© 2026 Pattalachinti VK et al. *JAMA Network Open*.

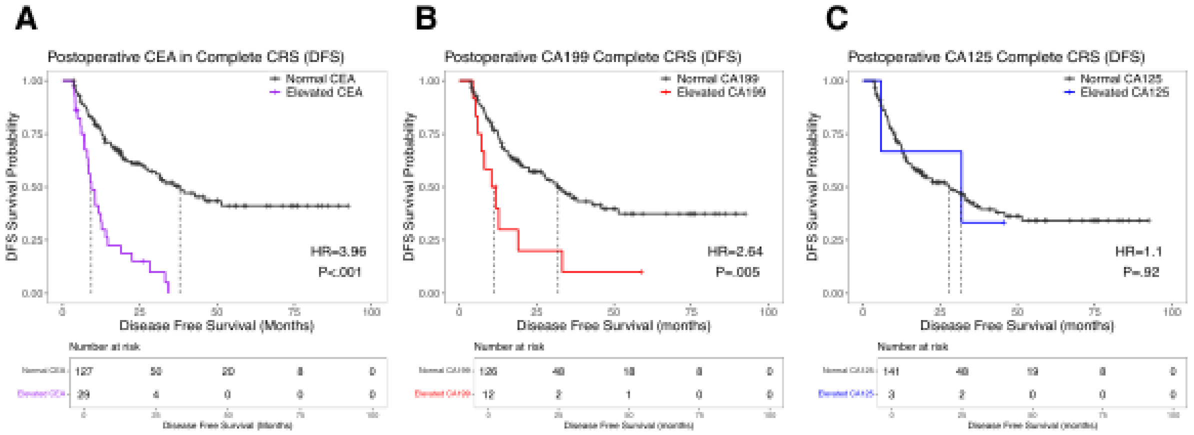

Supplemental Figure 10: Disease-free survival (DFS) based on postoperative tumor marker elevation. **10A**: KM plot of DFS stratified on postoperative CEA elevation. **10B**: KM plot of DFS stratified on postoperative CA199 elevation. **10C**: KM plot of DFS stratified on postoperative CA125 elevation. All HR are elevated vs normal. P-val by Cox proportional hazards test.

© 2026 Pattalachinti VK et al. *JAMA Network Open*.

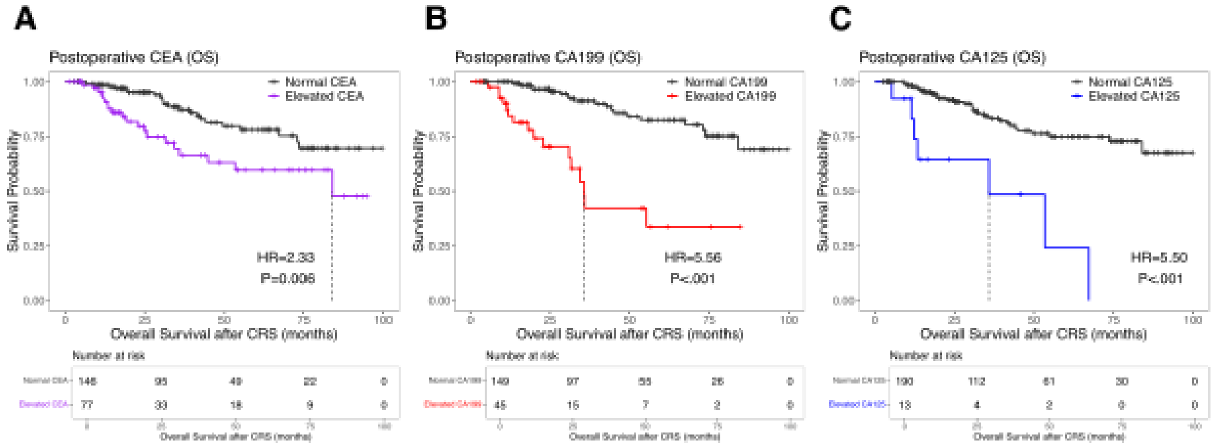

Supplemental Figure 11: OS based on postoperative tumor marker levels. **11A**: KM plot of OS stratified on postoperative CEA elevation. **11B**: KM plot of OS stratified on postoperative CA199 elevation. **11C**: KM plot of OS stratified on postoperative CA125 elevation. All HR are elevated vs normal. HR and P-val by Cox proportional hazards test.

© 2026 Pattalachinti VK et al. *JAMA Network Open*.

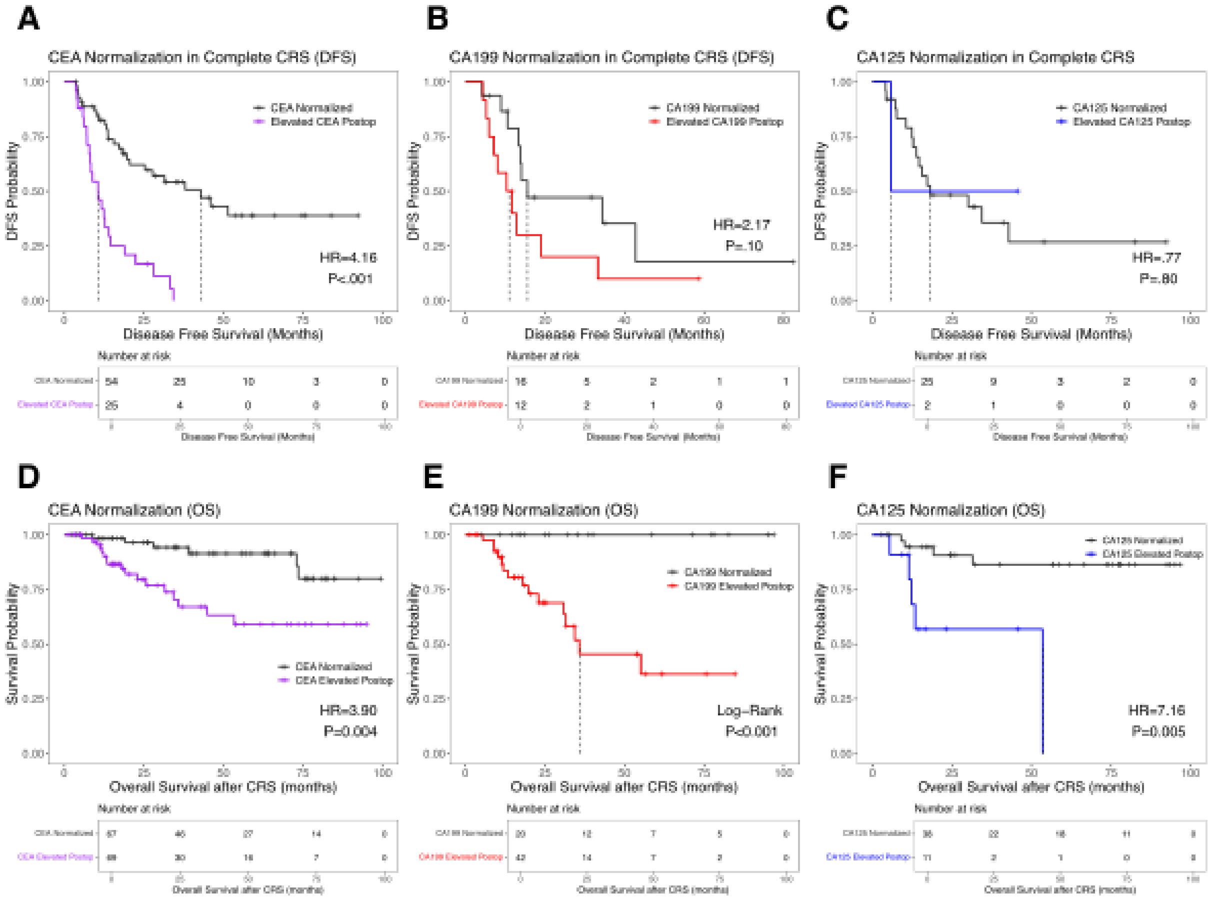

Supplemental Figure 12: Kaplan-Meier plots of disease-free survival (DFS) and overall survival (OS) in patients with elevated TM levels stratified by postoperative normalization. **12A**: KM plot of DFS stratified on postoperative CEA normalization in complete CRS. **12B**: KM plot of DFS stratified on postoperative CA199 normalization in complete CRS. **12C**: KM plot of DFS stratified on postoperative CA125 normalization in complete CRS. **12D**: KM plot of OS stratified on postoperative CEA normalization in incomplete CRS. **12E**: KM plot of OS stratified on postoperative CA199 normalization in incomplete CRS. P-val by log-rank test due to no events observed in the normalized group **12F**: KM plot of OS stratified on postoperative CA125 normalization in incomplete CRS. All HR are normal vs elevated. HR and P-val by Cox proportional hazards.

test or log-rank test where specified.

© 2026 Pattalachinti VK et al. *JAMA Network Open*.

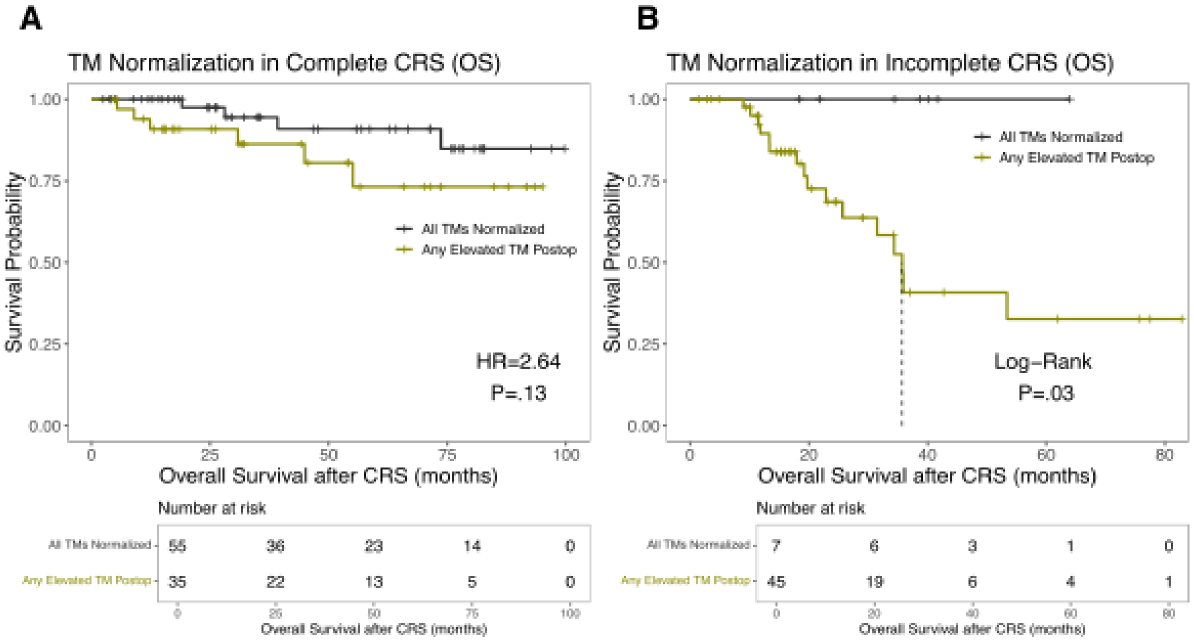

Supplemental Figure 13: Overall survival stratified by postoperative TM normalization. **13A**: In complete CRS. HR elevated vs normal. P-val by Cox proportional hazards test. **13B**: In incomplete CRS. P-val by Log-rank test.

© 2026 Pattalachinti VK et al. *JAMA Network Open*.

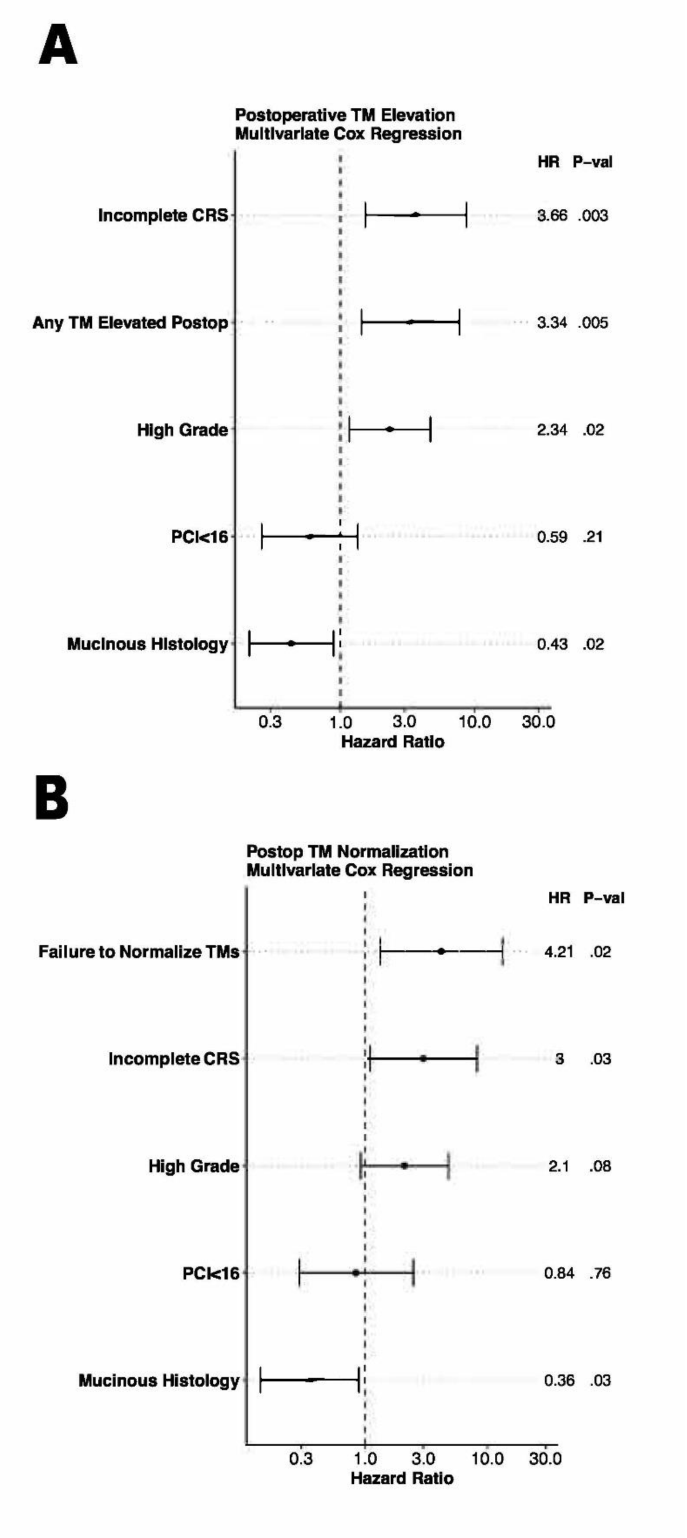

Supplemental Figure 14: Multivariate Cox proportional hazards models. **14A:** With postoperative TM elevation. **14B:** With postoperative TM normalization. HR elevated vs normal; HR and P-val by Cox Proportional Hazards Model.

© 2026 Pattalachinti VK et al. *JAMA Network Open*.
