# Supplementary material for: Appendiceal Adenocarcinoma Cytoreduction Outcomes and Perioperative Serum Tumor Marker Levels
Source: JAMA Netw Open. 2026 May 4;9(5):e2610569. doi: 10.1001/jamanetworkopen.2026.10569 (PMC13139952; doi:10.1001/jamanetworkopen.2026.10569)
Supplement: Supplement 2. — Data Sharing Statement [file jamanetwopen-e2610569-s002.pdf]

Data Sharing Statement

Pattalachinti. Appendiceal Adenocarcinoma Cytoreduction Outcomes and Perioperative Serum Tumor Marker Levels. *JAMA Netw Open*. Published May 04, 2026.
doi:10.1001/jamanetworkopen.2026.10569

Data
Data available: Yes
Data types: Deidentified participant data
How to access data: jshen8@mdanderson.org
When available: With publication

Supporting Documents
Document types: None

Additional Information
Who can access the data: researchers whose proposed use of the data has been approved per IRB
Types of analyses: any non-commercial IRB approved usage
Mechanisms of data availability: after approval of a proposal, and/or with a signed data access agreement
